# The zinc finger protein 3 of *Arabidopsis thaliana* regulates vegetative growth and root hair development

**DOI:** 10.3389/fpls.2023.1221519

**Published:** 2024-01-05

**Authors:** Dániel Benyó, Emese Bató, Dóra Faragó, Gábor Rigó, Ildikó Domonkos, Nitin Labhane, Laura Zsigmond, Melvin Prasad, István Nagy, László Szabados

**Affiliations:** ^1^ Instiute of Plant Biology, HUN-REN Biological Research Centre, Szeged, Hungary; ^2^ Department of Botany, Bhavan’s College, Mumbai, Maharashtra, India; ^3^ Institute of Biochemistry, HUN-REN Biological Research Centre, Szeged, Hungary; ^4^ SeqOmics Biotechnology Ltd, Mórahalom, Hungary

**Keywords:** *Arabidopsis thaliana*, zinc finger protein 3, gene overexpression, gene silencing, plant development, root hair, transcript profiling

## Abstract

**Introduction:**

Zinc finger protein 3 (ZFP3) and closely related C2H2 zinc finger proteins have been identified as regulators of abscisic acid signals and photomorphogenic responses during germination. Whether ZFP3 and related ZFP factors regulate plant development is, however, not known.

**Results:**

ZFP3 overexpression reduced plant growth, limited cell expansion in leaves, and compromised root hair development. The T-DNA insertion zfp3 mutant and transgenic lines with silenced ZFP1, ZFP3, ZFP4, and ZFP7 genes were similar to wild-type plants or had only minor differences in plant growth and morphology, probably due to functional redundancy. RNAseq transcript profiling identified ZFP3-controlled gene sets, including targets of ABA signaling with reduced transcript abundance. The largest gene set that was downregulated by ZFP3 encoded regulatory and structural proteins in cell wall biogenesis, cell differentiation, and root hair formation. Chromatin immunoprecipitation confirmed ZFP3 binding to several target promoters.

**Discussion:**

Our results suggest that ZFP3 and related ZnF proteins can modulate cellular differentiation and plant vegetative development by regulating the expression of genes implicated in cell wall biogenesis.

## Introduction

One of the challenges of current plant biology is to understand how gene regulatory networks control plant development or responses to environmental impacts. Zinc finger (ZnF) proteins belong to large gene families in plants, which can function as regulators of transcription, apoptosis, or cellular differentiation through interactions with DNA, RNA, or proteins (Englbrecht et al., 2004; Ciftci-Yilmaz and Mittler, 2008). In Arabidopsis, 176 C2H2-type ZnF proteins have been annotated, most of which are plant specific (Englbrecht et al., 2004). The 33 ZnF proteins of the C1-1i subclass have one ZnF domain and are among the most studied ZnF factors. Many of them are implicated in plant development, and can control cell differentiation and hormone signaling (Xie et al., 2019). Plant growth, leaf shape, and flowering are regulated by the closely related zinc finger protein 10 and 11 (ZFP10 and ZFP11), which cause dwarfism, abnormal leaf morphology, early flowering, and sterility when overexpressed in Arabidopsis (Dinkins et al., 2002; Dinkins et al., 2003). JAGGED (JAG) regulates cell cycle by promoting S phase entry, controls lateral organ development and patterning, and modulates genes that are involved in cell cycle progression, tissue polarity, and cell wall formation (Ohno et al., 2004; Schiessl et al., 2012). NUBBIN (NUB) is closely related to JAG and regulates stamen and carpel development together with JAG (Dinneny et al., 2006). The UPRIGHT ROSETTE (URO) is implicated in auxin homeostasis and has influence on auxin-related traits such as root formation (Sun et al., 2010). ZFP2 regulates flower abscission by forming a complex with the transcription factor AtDOF4.7, promoting the expression of genes of cell wall hydrolyzing enzymes (Wei et al., 2010). Several zinc finger proteins of the C1-1i subclass were shown to control trichome development and differentiation, including ZFP5, ZFP6, ZFP8, and the related Glabrous Inflorescence Stems (GIS), GIS2, and GIS3 factors. ZFP8 and GIS2 promote trichome formation through mediation of cytokinin signals and transmit gibberellin response together with GIS (Gan et al., 2007). Genetic analysis revealed that ZFP5 regulates GIS, GIS2, and ZFP8, and controls key factors of trichome initiation GLABROUS1 (GL1) and GL3 (Zhou et al., 2011). ZFP6 integrates gibberellin and cytokinin signals in regulation of trichome development upstream of ZFP5 (Zhou et al., 2013). GIS and GIS2 are direct targets of the closely related GIS3 in trichome initiation (Sun et al., 2015). The TRICHOME RELATED PROTEIN (TRP) was found to interact with ZFP5, blocking its binding on the ZFP8 promoter, which suppresses trichome initiation (Kim et al., 2018). ZFP5 was also shown to promote root hair development through mediation of ethylene and cytokinin signals (An et al., 2012). Enhancement of root hair development during potassium or phosphate starvation is also controlled by ZFP5, via activation of ETHYLENE INSENSITIVE 2 (EIN2) (Huang et al., 2020). On the other hand, the ZnF protein AtZP1 downregulates root hair initiation and elongation by suppressing a set of root hair-specific genes (Han et al., 2020). These reports show that ZnF proteins of the C1-1i subclass are versatile regulators of cell differentiation including trichome and root hair development.

Zinc finger protein 3 (ZFP3) has a single ZnF_C2H2 domain and has been identified due to its capacity to confer abscisic acid (ABA) insensitivity to seed germination of overexpressing Arabidopsis plants. Overexpression of the closely related ZFP1, ZFP4, ZFP6, and ZFP7 factors could also reduce ABA sensitivity, suggesting that they have partially redundant function. Moreover, ZFP3 was shown to enhance phytochrome-mediated red light signaling in hypocotyl elongation assays (Joseph et al., 2014). Influence of ZFP3 and the most closely related ZFP factors on vegetative stages of plant development was, however, not investigated. In this study, we analyzed the function of ZFP3 in plant growth and development, and showed that together with several closely related ZFPs, this ZnF protein modulates vegetative growth and is implicated in root hair formation.

## Materials and methods

### Gene cloning

Artificial microRNA (amiR) constructs were designed and generated according to the protocols of Weigelworld Web MicroRNA Designer: http://wmd3.weigelworld.org/cgi-bin/webapp.cgi?page=Home;project=stdwmd. Gateway recombination sequences were added to the 5’ end of the selected primer sequences for cloning. pRS300 plasmid was used as the template to perform PCR with the designed oligoes. PCR fragments were cloned into pDONR222.1 plasmid with BP clonase reaction, and verified by sequencing. Subsequently, inserts were moved to pTCO355 vectors with LR Clonase. Constructs were tested by sequencing and introduced into GV3101/pMP90 Agrobacterium strain for plant transformation.

### Plant material

For all experiments, Arabidopsis Col-0 ecotype was used except *abi4-104*, which has a Col(gl1) background. In the overexpressing lines, ZFP3 was tagged either by GFP or HA, and labeled by ZFP3g or ZFP3h, respectively. Estradiol-inducible (XVE-ZFP3h) and constitutively overexpressing (35S-ZFP3g, 35S-ZFP3h) lines were used. The ZFP3 promoter β-glucuronidase (pZFP3-GUS) expressing lines and the *zfp3* T-DNA insertion mutant line have already been described (Joseph et al., 2014). *zfp1*, *zfp4*, and *zfp8* T-DNA insertion lines were acquired from the SAIL and SALK collections, respectively. Standard PCR with genome- and T-DNA-specific primers were used to confirm genotype of the mutants and identify homozygous lines (**Supplementary Figure 2**). Agrobacterium-mediated Arabidopsis transformation with new constructs was made as described (Rigó et al., 2016). Seeds of infiltrated plants were collected and transgenic plants were identified by BASTA selection. A total of 24 independent lines were tested for silencing of the target genes, and three representative lines were used for subsequent experiments. Arabidopsis lines used in this study are listed in **Supplementary Table 1**.

### Plant growth measurements

For *in vitro* growth assays, seeds were surface sterilized and germinated on ½ MS medium containing 0.5% sucrose and 0.8% agar, pH 5.7. Plants were grown in growth chambers under 150 µmol m^−2^ s^−1^ photon flux density at 8/16 h light/dark cycle, at 22/20°C temperature under light/dark conditions. For gene induction, 5-day-old seedlings were transferred to media supplemented with 5 µM β-estradiol or 2.5 µM ABA or both. ABA sensitivity was evaluated by measuring rosette sizes of 14-day-old plants by applying custom-made ImageJ macro (**Supplementary Method**). Root hair lengths of 7-day-old ZFP-overexpressing or silenced plants were measured manually by ImageJ’s default measuring tools. The number of root hairs was counted on the same plants. Root hair numbers and lengths were determined on at least eight roots of a genotype.

### Plant phenotyping

Plant growth was monitored by measuring rosette sizes with an automatic plant phenotyping platform (PantScreen™ Compact System, Photon System Instruments, Brno, Czech Republic). Forty plants were analyzed for each genotype. Plants were germinated and grown under controlled growth conditions under a 12 h/12 h 22°C/20°C light/dark cycle with an irradiance of 200 µmol m^−2^ s ^−1^. RGB imaging was made for 5 days on daily intervals from 27 to 31 days after germination using a top view GigE PSI RGB camera (Sony IMX253LQR-c). RGB image processing was made with a PlantScreen™ Data Analyzer (PSI, Czech Republic). Further data curation and analysis was performed with “ MVApp” statistical application (Julkowska et al., 2019). Before data analysis, samples below (*R* ^2^ < 0.8) threshold and identified outliers (1.5 × interquartile range) were removed from the curated dataset. During standard statistical analysis, the rosette size (mm^2^) did not show normal distribution or equal variance; therefore, the effect of genetic variation was examined by performing analysis of variance (Kruskal–Wallis) with pairwise Wilcoxon test/Mann–Whitney test of significance (*p*-value < 0.05).

### RNAseq transcript profiling, plant treatment

Transcript profiling was made with ZFP3-overexpressing Arabidopsis plants carrying the estradiol-inducible ZFP3 and ZFP7 constructs (Joseph et al., 2014). For RNAseq analysis, Col-0 and XVE-ZFP3h1 and XVE-ZFP7 plants were grown as defined above. Control and short-term estradiol-treated plants were grown on ½ MS medium for 2 weeks, while plants subjected for long-term induction were cultured on medium supplemented with 5 µM β-estradiol. Two weeks-old plants were sprayed with water (Control), 5 µM β-estradiol (long induction), or 50 µM β-estradiol solution for 6 h (short induction) before sample collection. Total RNA was isolated from plants as described (Yaffe et al., 2012). The extraction buffer’s composition was modified by using 6 M guanidine thiocyanate, 20 mM MES hydrate, and 20 mM EDTA, to prevent rapid crystallization at room temperature. Five micrograms of total RNA was treated with Invitrogen’s TURBO DNA-free Kit, and 1 μg of DNase-treated RNA was used for transcript sequencing. Three biological replicates were made.

### Sequencing and data analysis

Libraries for RNA-seq were prepared using NEBNext® Ultra™ RNA library preparation kit, following the manufacturer’s instructions, and sequenced on Illumina NovaSeq 6000 platform using 2×150 bp, with a mean depth of 10^7^ reads/sample. The raw reads were adapter and Q30 quality trimmed with Trimmomatic (Bolger et al., 2014), and quality assessed using FastQC (http://www.bioinformatics.babraham.ac.uk/projects/fastqc/). Pre-processed reads were mapped to the reference genome TAIR10 using the spliced mapper HISAT2 (Kim et al., 2019), then transcripts were assembled and quantified using StringTie (Pertea et al., 2015). Differentially expressed genes were identified with edgeR (Robinson et al., 2010). Functional enrichment analysis was performed with g:Profiler (https://biit.cs.ut.ee/gprofiler/gost) (Raudvere et al., 2019), together with TAIR’s tool for GO Term Enrichment (https://www.arabidopsis.org/tools/go_term_enrichment.jsp). For visualization and GO term overrepresentation analysis, BiNGO was implemented in Cytoscape (Shannon et al., 2003).

### Gene expression and qRT-PCR studies

For qRT-PCR analysis, two weeks-old Arabidopsis plants were grown under the same conditions as described for growth measurements. Total RNA was isolated as described for RNAseq analysis. One microgram of DNase-treated RNA was used for cDNA synthesis using the High-Capacity cDNA Reverse Transcription Kit (Applied Biosystems). qRT-PCR was performed on 3 μl of 20× diluted cDNA templates using 5× HOT FIREPol EvaGreen qPCR Mix Plus (Solis Biodyne) in a final volume of 8 μl, and Bio-Rad CFX96 Touch Deep Well Real-Time PCR System. Mean values of polyubiquitin 10 (*UBQ10, AT4G05320*) and glyceraldehyde-3-phosphate dehydrogenase C2 (*GAPC2, AT1G13440*) Ct were used as internal reference. Normalized relative transcript levels were obtained by the 2^−ΔΔCt^ method (Livak and Schmittgen, 2001). Experiments were repeated with three biological replicates. Oligonucleotides used in this study are listed in **Supplementary Table 12**.

### Chromatin immunoprecipitation

Plants were grown and treated with estradiol as described above. Cross -linking, isolation of nuclei, sonication, and final purification of DNA were performed as previously described (Andrási et al., 2019). Immunoprecipitation of genomic DNA fragments with HA epitope-tagged ZFP3 was performed by using a Miltenyi Biotec µMACS™ HA Isolation Kit according to the manufacturer’s instructions. Putative ZnF binding sites were identified with the online tool AthaMap (http://www.athamap.de, **Supplementary Table 11**). PCR amplification of target sequences was made with qPCR Solis BioDyne HOT FIREPol® EvaGreen® qPCR Mix, using primers specific to the selected promoter regions (**Supplementary Table 12**). Calculation of promoter binding efficiency was made by normalization of ChIP-qPCR data as previously described (Solomon et al., 2021). Fold enrichment was calculated using a UBQ10 promoter region as reference.

### Scanning electron microscopy

Samples were vacuum infiltrated and fixed with 100% methanol for 20 min, dehydrated in 100% ethanol for 30 min, and then in fresh 100% ethanol overnight. Next day, samples were critical point dried, mounted on SEM stubs, and observed in a JEOL JSM-7100F/LV scanning electron microscope in low-vacuum mode. High-contrast cell outlines in the uncoated leaves were imaged according to Talbot and White (2013) by detecting backscattered electrons at 15 kV accelerating voltage and 35 Pa pressure in the specimen chamber. SEM images were analyzed with the updated version of the PlantSize software (Faragó et al., 2018). For quantitative measurements, at least 6 images were analyzed for each genotype and condition (MS medium-grown or soil-grown plants) of leaf surface adaxial and abaxial epidermis. At least 50 cells were measured in each SEM image. Area and circularity values were measured and data were processed with MS Excel software.

## Results

### ABA responses of ZFP3-overexpressing plants

**Figure 1:**
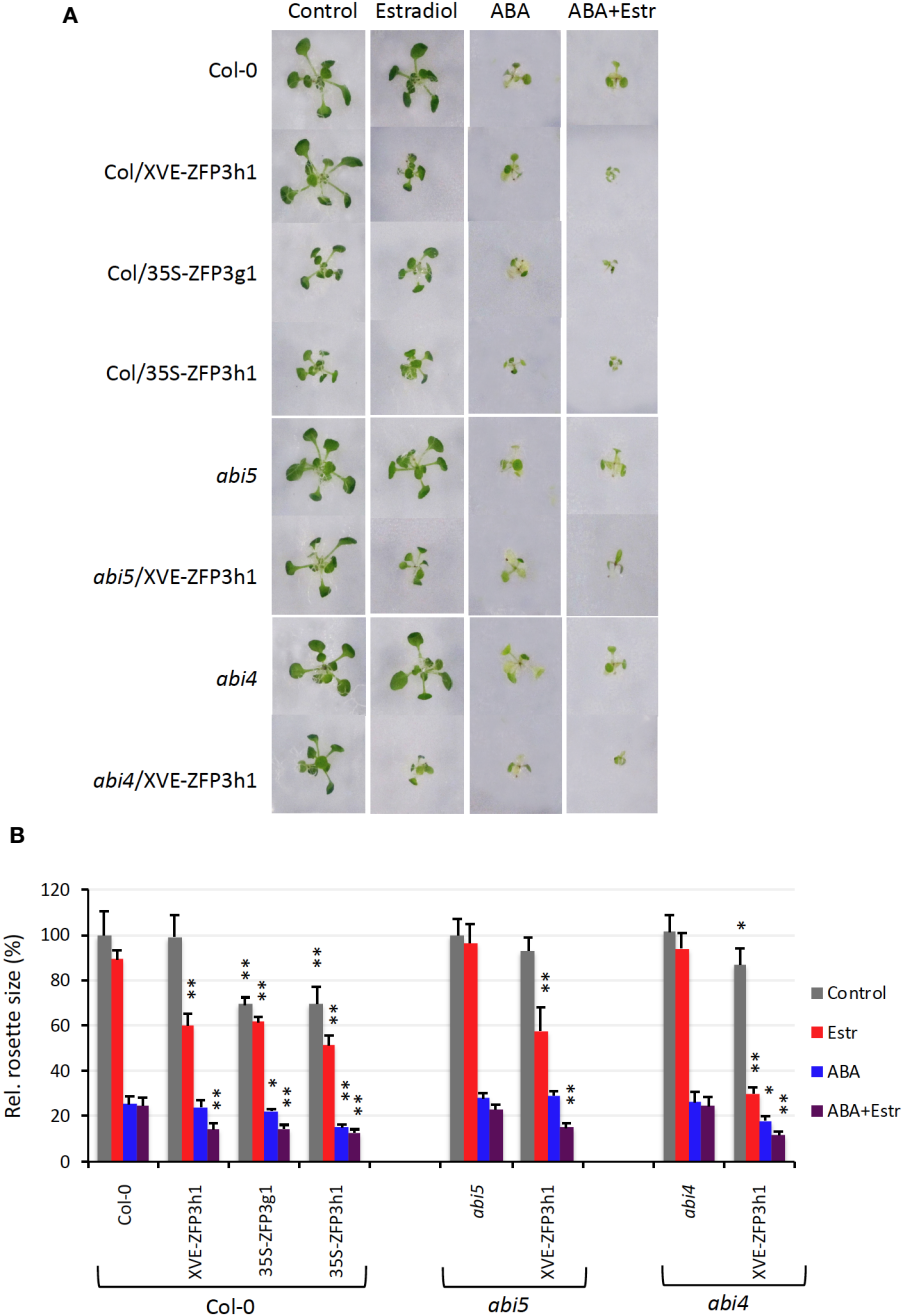
ABA sensitivity of ZFP3-overexpressing plants grown on MS medium. **(A)** Typical rosettes of 14-day-old plants grown on media supplemented with 5 μM estradiol or 25 μM ABA or both for 9 days. Col-0: wild type, Col/XVE-ZFP3h1: estradiol-inducible ZFP3 in Col-0 background. Col/35S-ZFP3g1, Col/35S-ZFP3h1: constitutively expressing, GFP, or HA-tagged ZFP3 controlled by the CaMV35S promoter in Col-0 background. *abi5*, *abi4*: ABA insensitive 5 and ABA insensitive 4 mutants, abi5/XVE-ZFP3h1, abi4/XVE-ZFP3h1: estradiol-inducible ZFP3 in *abi5* or *abi4* backgrounds, respectively. **(B)** Rosette sizes of 14-day-old plants determined on the base of their green area. Relative values are shown where 100% corresponds to untransformed plants at control conditions. Error bars indicate standard error. Significant differences between rosette sizes of ZFP3-overexpressing and non-transformed plants (Col-0, abi5, abi4) are shown by * *p* < 0.05 and ** *p* < 0.01 (*N* = 10) (*t*-test).

ZFP3 was identified due to its capacity to reduce ABA sensitivity of seed germination, suggesting that this factor is a negative regulator of ABA signals. Overexpression of similar ZFP factors could also confer ABA insensitivity to germinating seeds, indicating a certain degree of functional redundancy in closely related ZFPs (Joseph et al., 2014). To study ABA responses in growing plants, gain- and loss- of-function approaches were employed. To test possible epistasis with known ABA signaling factors, ABA responses of XVE-ZFP3h1 plants were tested not only in Col-0, but also in the ABA-insensitive *abi4* and *abi5* mutant backgrounds (Finkelstein et al., 1998; Finkelstein and Lynch, 2000; Joseph et al., 2014). Plant growth (rosette size and root length) was monitored on media supplemented with ABA or estradiol or both. On standard growth conditions, rosette sizes of all genotypes were similar to Col-0, except the 35S-ZFP3g1 and 35S-ZFP3h1 plants, which constitutively overexpressed the GFP or HA-tagged ZFP3. Such ZFP3-overexpressing plants had approximately 35% smaller rosettes than wild-type ones. On estradiol-containing media Col-0, *abi4* and *abi5* plants were similar to plants grown on control conditions, while plants with XVE-ZFP3h construct were 30% to 60% smaller. ABA (25 μM) reduced rosette sizes of all plants by 70%, including *abi4* and *abi5*, which are otherwise ABA insensitive during germination. When Col-0, *abi4*, and *abi5* plants were grown in the presence of ABA and estradiol, rosette sizes were similar to ABA-treated plants, while growth was more severely reduced with the XVE-ZFP3h construct. Similar to the XVE-ZFP3h1 plants, rosette growth of the 35S-ZFP3g1 and 35S-ZFP3h1 lines was also inhibited by ABA (**Figure 1**).

Roots of XVE-ZFP3h1 plants were 15% to 20% smaller than non-transgenics in the presence of estradiol irrespective of the genetic background (Col-0, *abi4*, or *abi5*). Root elongation was reduced by increasing the concentration of ABA, which was further reduced by ZFP3 overexpression. Root lengths were similarly inhibited by ZFP3 overexpression in Col-0 wild type and *abi4* or *abi5* mutants (**Supplementary Figure 1**). These results show that ZFP3 does not confer ABA insensitivity to overexpressing plants during post-germination development. ZFP3 overexpression, however, reduced vegetative growth and with ABA treatment led to severe growth inhibition in all genetic backgrounds. The additive nature of ABA treatment and ZFP3 overexpression on growth reduction suggests that ZFP3 controls vegetative growth parallel to ABA regulation.

### ZFP3 modulates growth and development

Reduced rosette and root sizes suggested that ZFP3 has a negative influence on plant growth. This observation prompted us to investigate rosette development of soil-grown plants using transgenic lines with constitutively overexpressing ZFP3 constructs, T-DNA insertion mutants, and silenced lines in more detail. For loss- of-function analysis, T-DNA insertion mutants *of ZFP1* (SAIL_657_E10), *ZFP3* (GK-177E02), *ZFP4* (SALK_038923), and *ZFP8* (SALK_045674) genes were studied. Testing the expression of these genes in the mutants however showed that they had transcript levels comparable to wild-type plants (**Supplementary Figure 2**). Sequence analysis of the *zfp3* mutant revealed that an open reading frame exists in the 3’ direction of the T-DNA insertion site, which might encode a truncated ZFP3 protein. The truncated N-terminal ZFP3 fragment, however, cannot be functional as the ZnF domain is deleted. To study the loss- of-function phenotypes of ZFP genes, silenced lines were subsequently generated by artificial microarray technology using amiR constructs driven by the constitutive CaMV35S promoter. The targeted *ZFP1, ZFP3, ZFP4*, and *ZFP7* genes were silenced with variable efficiency, as their expression in the amiR lines ranged between 18% and 36% of the wild type (**Supplementary Figure 3**). Silenced lines with the lowest transcript levels were used for subsequent analysis.

**Figure 2:**
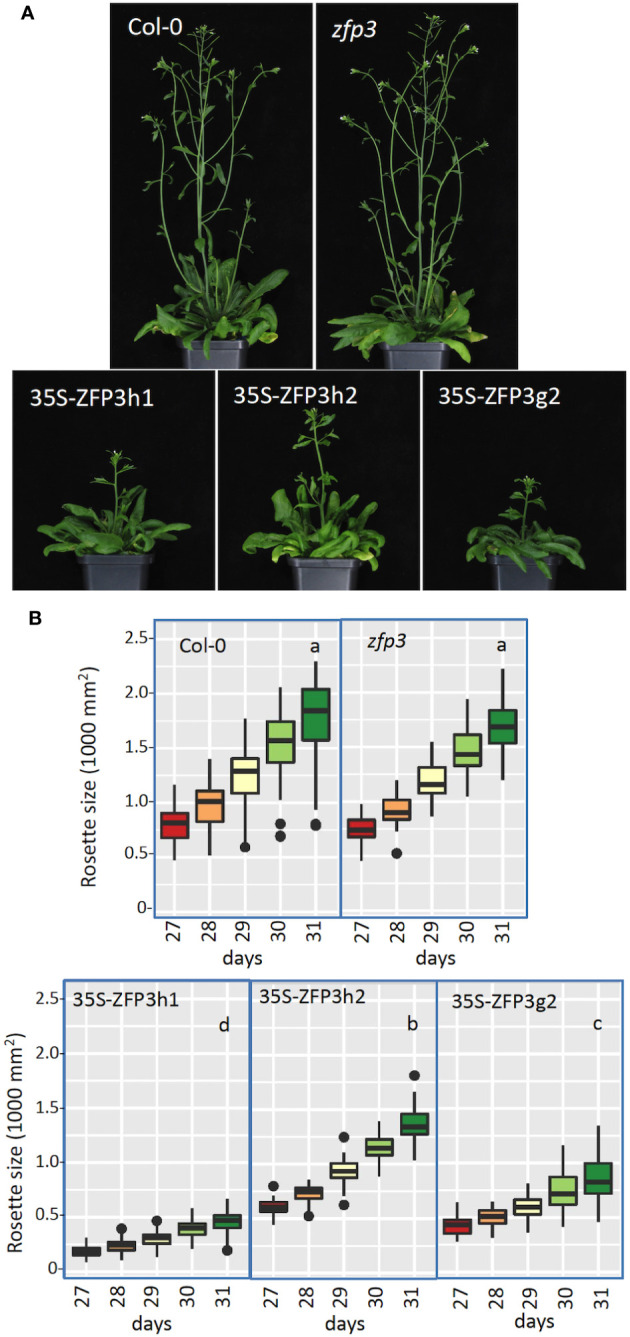
Phenotype of *zfp3* mutant and ZFP3-overexpressing plants in soil. **(A)** Images of a typical Col-0 plant, *zfp3* mutant, and transgenic plants with 35S-ZFP3 gene constructs. **(B)** Change in rosette areas of plants 27 to 31 days after germination. Rosette sizes were determined by automatic phenotyping using RGB imaging and measuring green pixel areas. Analysis of variance was performed (Kruskal–Wallis) with pairwise Wilcoxon test/Mann–Whitney test of significance. Different letters indicate significant differences between the genotypes (*N* = 40, *p*-value < 0.05).

Growth and morphology of transgenic plants overexpressing HA or GFP-tagged ZFP3 (35S-ZFP3h1, 35S-ZFP3h2, and 35S-ZFP3g2), amiR silenced lines, and the *zfp3* insertion mutant were monitored under controlled conditions using an automatic phenotyping platform. ZFP3-overexpressing plants frequently displayed growth defects, had wrinkled leaves, and flowered with some delay than Col-0 plants, while the *zfp3* mutant was similar to wild type (**Figure 2A**). Image analysis revealed that rosette sizes of soil-grown ZFP3-overexpressing plants were 25% to 70% smaller than Col-0 plants, depending on the transgenic line analyzed, while the *zfp3* insertion mutant was similar to wild type (**Figure 2B**, **Supplementary Figure 4**). amiR-mediated silencing of *ZFP1, ZFP3, ZFP4*, and *ZFP7* genes had no dramatic consequence on plant phenotype and growth. Plant morphology was similar to Col-0 and growth was comparable or slightly reduced when compared to wild type (**Supplementary Figures 4**, **5**).

To test possible functional redundancy in the ZFP subfamily of ZnF factors, T-DNA insertion mutants and amiR lines were crossed in different combinations to generate double mutants and silenced lines. Crossed double and triple mutants were similar to wild-type plants. When two ZFP genes were simultaneously silenced in crossed transgenic lines (ZFP1+ZFP3, ZFP1+ZFP4, and ZFP4+ZFP7), plant growth and morphology were similar to plants silenced with single ZFP genes and wild-type plants (not shown).

**Figure 3:**
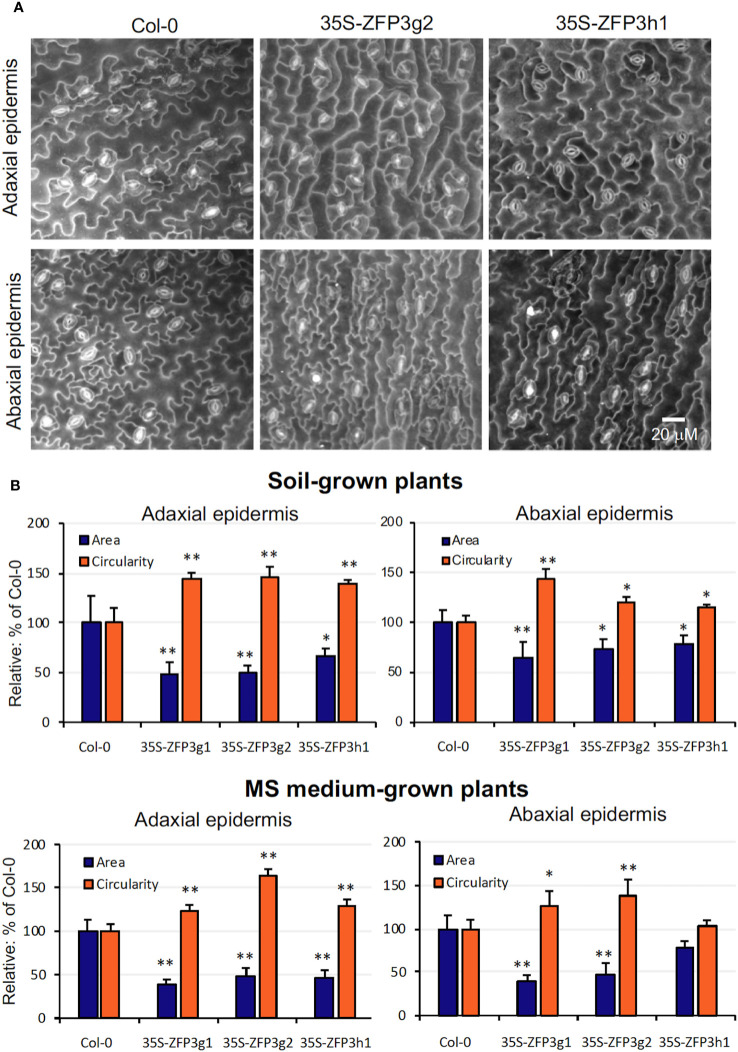
Size and morphology of leaf epidermal cells of ZFP3-overexpressing plants. **(A)** Typical scanning electron microscopic images of leaf adaxial and abaxial surfaces of Col-0 and two 35S-ZFP3 plants. **(B)** Quantitative analysis of epidermal cells. Average cell area and circularity were determined with PlantSize software and were normalized to values obtained on wild-type plants (=100%). Averages obtained on 8 to 12 images are shown; on each image, at least 50 cells were measured. Error bars on diagrams indicate standard deviation; significant differences between Col-0 and transgenic plants are shown by *p < 0.05 and ** *p* < 0.01 (*N* = 40) (*t*-test).

The semi-dwarf phenotype of ZFP3-overexpressing plants can be the result of defects in cell division, elongation, and differentiation. Scanning electron microscopy was used to study the size and shape of leaf epidermal cells in ZFP3-overexpressing and wild-type plants. Cell area and circularity were calculated to characterize differences in cell size and shape, respectively. Although great variability of epidermal cells was present in all genotypes, cells of 35S-ZFP3h1, 35S-ZFP3g1, and 35S-ZFP3g2 plants were generally smaller than wild-type ones, and had a less complex shape (**Figure 3A**). Average cell areas of 35S-ZFP3 cells were 30% to 60% smaller than wild type, irrespective if grown on soil or on ½ MS medium. Lobe formation of these cells was also compromised, leading to more simple cell forms, indicated by higher circularity. While cell areas correlated with cellular perimeters, circularity had negative correlation with cell size (**Supplementary Figures 6**, **7**). Such correlation could be observed for all genotypes to different degrees. The difference between the wild-type and transgenic plants was, however, displayed by a smaller average size and higher circularity of ZFP3-overexpressing epidermal cells (**Figure 3B**).

### Hundreds of genes are regulated by ZFP3

In order to identify genes that are regulated by ZFP3 during vegetative growth, whole genome transcript profiling was performed with Col-0 and XVE-ZFP3h1 plants, using RNAseq technology. As the *zfp3* mutant and the silenced zfp3amiR plants were not very different from wild type, they were not included in such transcript analysis. Estradiol-inducible ZFP3 overexpression was employed and samples were collected after 6 h and continuous estradiol treatments. The chemically controlled expression system permitted the identification of gene sets activated or repressed by short and continuous ZFP3 overexpression. RNAseq reads were mapped to 38,681 loci, out of which 22,362 genes had detectable transcript levels and were analyzed (**Supplementary Table 2**). A total of 1,652 genes had at least 2.5 times difference in transcript abundance between Col-0 and XVE-ZFP3 plants. Six hours of estradiol treatment repressed and induced 641 and 571 genes, respectively. A total of 308 and 421 genes had reduced or enhanced transcript levels after long estradiol exposure, respectively. These results indicate that 73% of the ZFP3-regulated genes responded within 6 h of ZFP3 activation, while continuous overexpression modulated 44% of all regulated genes (**Supplementary Figure 8**, **Supplementary Tables 2**-**6**).

Gene ontology (GO) classification revealed that many genes that were downregulated by short ZFP3 overexpression are implicated in cell wall biosynthesis and organization, root hair differentiation and elongation, and response to oxidative stress and to water deprivation. Continuous ZFP3 overexpression repressed fewer genes and GO terms included aging, response to hypoxia, and secondary metabolism. Most GO Cellular component categories included protein located in cell wall or extracellular region (**Supplementary Figures 9**, **10**, **Supplementary Table 7**). GO terms related to glucosinolate biosynthesis and sulfur starvation were overrepresented among the genes induced by 6-h ZFP3 overexpression, while the only GO category of the ZFP3-induced genes of continuous overexpression was stomatal complex development, with low enrichment (**Supplementary Figures 10**, **11**, **Supplementary Table 7**).

**Figure 4:**
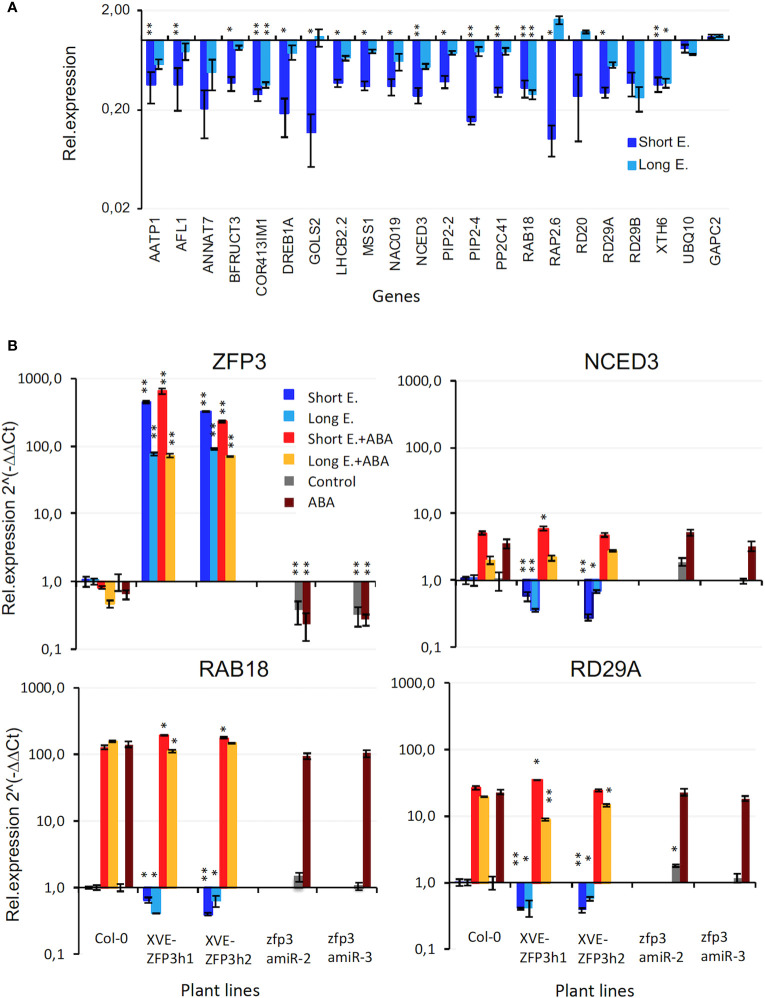
Expression of dehydration and ABA-responsive genes in XVE-ZFP3h1 plants. **(A)** Change in transcript levels of 20 genes of GO term “Response to water deprivation” as determined by RNAseq. Relative transcript levels are shown in log scale, compared to transcript levels of Col-0 wild type plants (=1). **(B)** Expression of ABA and dehydration induced *NCED3*, *RAB18*, and *RD29A* genes in ZFP3-overexpressing (XVE-ZFP3h1, h2) and silenced (zfp3amiR-2, -3) plants, determined by qRT-PCR. Relative expression levels are shown in log scale (Col-0 = 1). Short E. indicates 6-h induction of ZFP3, Long E. corresponds to plants grown on estradiol-containing medium, ABA refers to 6 h of 25 μM ABA treatment. Error bars on diagrams indicate standard deviation (*N* = 3), significant differences between transcript levels of Col-0 and transgenic plants are shown by *p < 0.05 and **p < 0.01 (*t*-test).

RNAseq identified a set of ZFP3-suppressed genes that respond to water deprivation and ABA, with three to eight times lower transcript levels in XVE-ZFP3h1 plants than in Col-0 (**Figure 4A**). Repression was stronger after 6 h of estradiol than with continuous treatment.

Differences in gene expression were verified by transcript analysis of selected genes in independent experiments, using quantitative RT-PCR. ZFP3 transcript levels were two to three orders of magnitude higher in estradiol-treated XVE-ZFP3h1 and XVE-ZFP3h2 plants than in Col-0 wild type, with higher expression after 6 h of estradiol treatment than with continuous treatment. ZFP3 transcript levels were reduced by 65% to 75% in zfp3amiR-2 and zfp3amiR-3 silenced lines. Transcript levels of ZFP3 were not influenced by ABA (**Figure 4B**). Expression of known dehydration and ABA-responsive genes was subsequently tested by qRT-PCR analysis of *NCED3*, *RAB18*, and *RD29A* genes in Col-0 wild type, XVE-ZFP3, and zfp3amiR lines. ZFP3 overexpression reduced transcript levels of all stress-induced genes by 40% to 70%, while ZFP3 silencing had only a marginal effect on them. ABA-dependent activation of *NCED3*, *RAB18*, and *RD29A* genes was similar in wild-type, ZFP3-overexpressing, or silenced plants (**Figure 4B**). These results confirmed that stress- and ABA-induced genes *NCED3*, *RAB18*, and *RD29A* can be repressed by ZFP3 overexpression, but not influenced by silencing. Induction of these genes by ABA was apparently not affected by ZFP3.

### ZFP3 regulates cell wall-related genes

**Figure 5:**
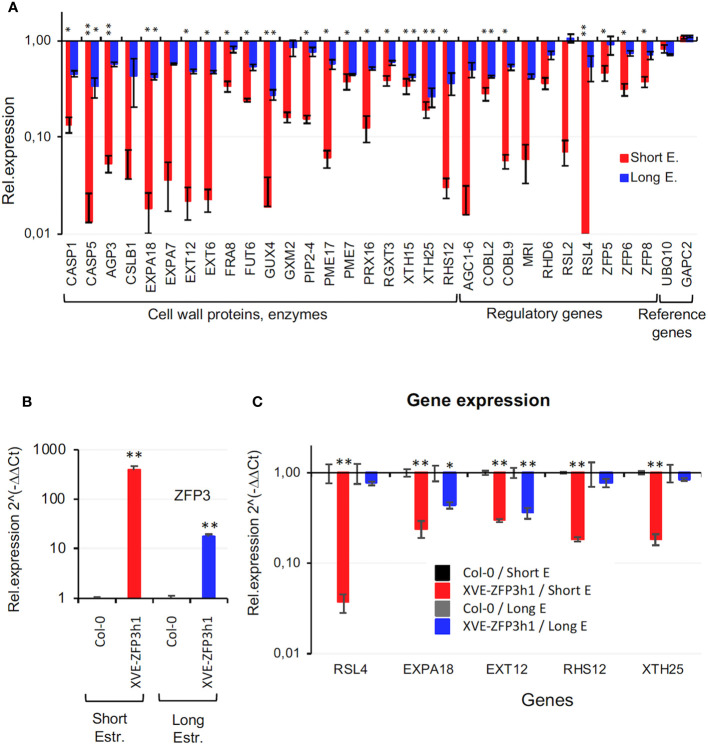
Expression of Arabidopsis genes implicated in cell wall formation, root hair growth and modification. **(A)** Transcript levels of 30 cell wall and root hair-related genes identified by RNAseq. Relative transcript levels are shown in log scale, compared to wild-type plants (Col-0 = 1). **(B)** Expression of ZFP3 in Col-0 wild type and the XVE-ZFP3h1 line used for subsequent qRT-PCR and ChIP experiments. Plants were treated with short and long estradiol treatment before sample collection. **(C)** qRT-PCR analysis of five cell wall and root hair-related genes: *RSL4*, *EXPA18, EXT12, RHS12*, and *XTH25* in Col-0 and XVE-ZFP3h1 plants. Relative transcript levels are shown where 1 corresponds to Col-0. Error bars indicate standard deviation (*N* = 3), significant differences between transcript levels of Col-0 and transgenic plants are shown by *p < 0.05 and **p < 0.01 (*t*-test).

GO analysis revealed that many genes that are implicated in cell wall biogenesis and modification are overrepresented in ZFP3-repressed gene sets, especially after 6 h of ZFP3 induction (**Supplementary Figure 9**, **Supplementary Table 3**). Such genes encoded enzymes in cell wall biogenesis including cellulose synthase (CSLB), xyloglucan endotransglucosylase/hydrolases (XTH), and hydroxyproline-rich proteins such as extensins (EXT), expansins (EXPA), pectine methylestherases (PME), fucosyl transferases (FUT), and casparian strip membrane domain proteins (CASP). Regulatory genes such as *AGC1-6, COBL9, MRI, RSL2*, and *RSL4*, which control cell differentiation, trichome, or root hair development, were also repressed by ZFP3 while *UBQ10* and *GAPC2* genes, used as references in qRT-PCR experiments, were not affected (**Figure 5A**).

In order to verify transcript profiling data in independent experiments, transcript levels of selected genes implicated in cell wall biogenesis and root hair formation were analyzed in Col-0 and XVE-ZFP3h1 plants, using qRT-PCR (**Figure 5B**). Expression of *RSL4*, *EXPA18, EXT12, RHS12*, and *XTH25* genes was considerably repressed in the XVE-ZFP3h1 line after 6 h of estradiol treatment, while continuous ZFP3 overexpression had less effect on them (**Figure 5C**). These results were comparable to RNAseq data and demonstrated that a set of genes implicated in cell wall biogenesis or modification are indeed downregulated by ZFP3. Most striking repression was observed on *RSL4*, a bHLH-type TF gene that controls root hair initiation (Datta et al., 2015) (**Figures 5A, C**). Transcript levels of the cell wall-related genes were subsequently compared in two independent ZFP3-overexpressing and two silenced lines. Estradiol-dependent activation of ZFP3 generated similar changes in the transcript profiles of the tested genes in both XVE-ZFP3h lines, while gene silencing has no or only minor effect on their expression (**Supplementary Figure 12**). These results suggest that ZFP3 may function as a suppressor of a group of genes implicated in cell wall biosynthesis and root hair formation, including transcription factors that control these processes.

**Figure 6:**
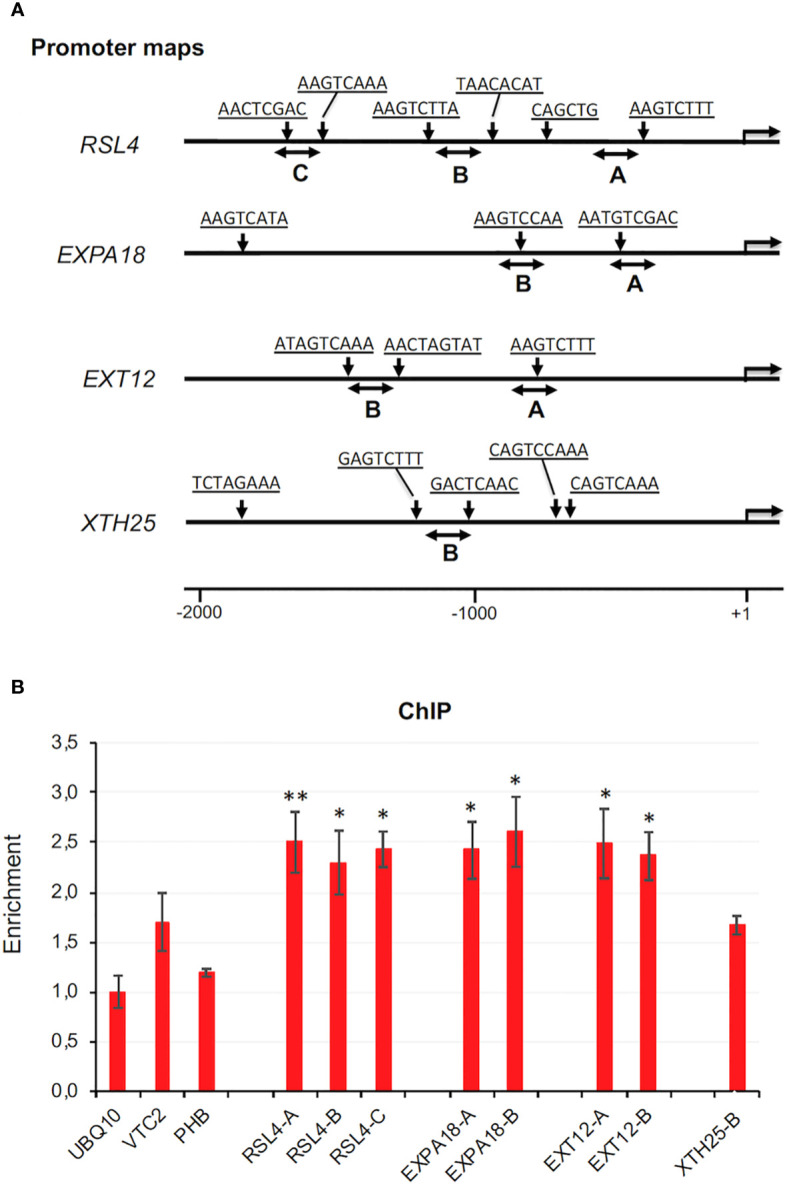
ChIP analysis of *ZFP3* promoter binding. **(A)** Schematic map of the 5’ regions of *RSL4*, *EXPA18, EXT12*, and *XTH25* genes. Arrowheads indicate positions of sequence motifs predicted to interact with ZnF factors (sequences are listed in **Supplementary Table 11**). Double arrows indicate regions amplified by qPCR in ChIP experiment. **(B)** Enrichment of the indicated promoter regions by qPCR after ChIP. Relative values shown where 1 corresponds to amplification of a UBQ10 coding region, which has no ZnF binding motif. Error bars on diagrams indicate standard deviation (*N* = 3). Significant differences between enrichment of UBQ10 and the tested region are shown by *p < 0.05 (*t*-test).

To test whether ZFP3 directly regulates the expression of these genes, a chromatin immunoprecipitation (ChIP) experiment was performed with promoter sections of selected ZFP3-regulated genes, including the transcription factor *RSL4*. Promoter regions of 2 kb length were analyzed to identify putative ZnF binding sequence motifs and were selected for ChIP-qPCR (**Figure 6A**). The promoter of the *UBQ10* gene had no such sequence element and was used as reference. In wild-type plants, *ZFP3* expression is quite low, which may encumber ChIP analysis with its native promoter. For this reason, and because estradiol-dependent activation of ZFP3 leads to striking differences in transcript profiles, the XVE-ZFP3h1 plants were used for ChIP analysis and plants were treated with estradiol for 6 h before sample collection. For ChIP experiments, 0.5 μM estradiol was used, which was one order of magnitude lower than the concentration routinely employed for gene expression or phenotype analysis. ChIP-PCR was performed with three promoter regions of RSL4 and two regions of EXPA18 and EXT12 and XTH25 genes (**Figure 6A**). Three genomic regions were selected as controls, where ZnF binding cis sequence elements were not predicted (promoters of *UBQ10, VTC2*, and *PHB*). When normalized ChIP-qPCR data were compared, significant enrichments were found with promoter regions of RSL4, EXPA18, and EXT12 genes, but not with control genes that lacked ZnF binding elements (**Figure 6B**). These data suggest that ZFP3 can interact with promoter regions of certain target genes and downregulate their transcription through direct promoter binding.

### Root hair formation is reduced by ZFP3 overexpression

**Figure 7:**
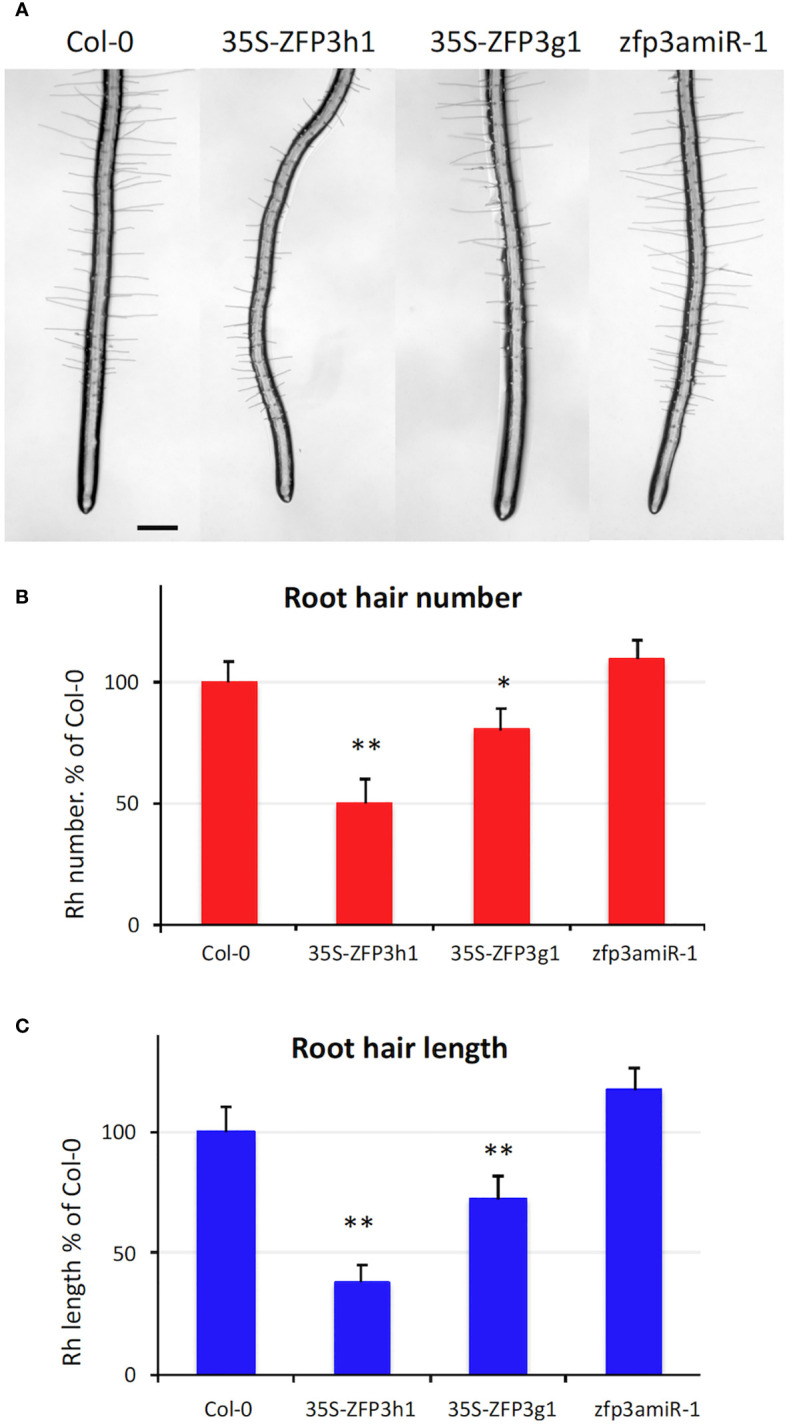
ZFP3 regulates root hair formation. **(A)** Typical roots of 35S-ZFP3h1, 35S-ZFP3g1, and zfp3amiR-1 plants grown on the surface of MS culture medium (scale bar = 200 μm). **(B)** Number of root hairs in a 2-mm section of wild-type, ZFP3-overexpressing, or silenced plants. **(C)** Root hair lengths of ZFP3-overexpressing or silenced plants. Relative values are shown, normalized to root hair numbers or lengths of Col-0 wild-type plants. Root hair lengths were measured with ImageJ. Error bars on diagrams indicate standard error (*N* = 40), significant differences between Col-0 and transgenic plants are shown by *p < 0.05 and **p < 0.01 (*t*-test).

RNAseq analysis demonstrated that many genes that are implicated in cell wall biogenesis and root hair formation were downregulated by ZFP3 overexpression (**Figure 5**, **Supplementary Figures 9**, **12**, **Supplementary Table 3**). Such data prompted us to investigate the effect of ZFP3 on root hair development. Wild-type, ZFP3-overexpressing, and *ZFP3* silenced plantlets were grown on vertical plates to monitor root hair formation. Root hairs were less abundant on 35S-ZFP3g1 and 35S-ZFP3h1 roots (**Figure 7A**). The number of root hairs was 50% to 75% lower in ZFP3-overexpressing roots when compared to wild type (**Figure 7B**). Root hairs of these ZFP3-overexpressing plants were 30% to 60% shorter than in Col-0 (**Figure 7C**). Root hairs of zfp3amiR-1 plants were similar to Col-0 roots (**Figures 7A–C**). Defects in root hair formation and lengths suggest that root hair development can be compromised by ZFP3 overexpression.

Root hair formation was monitored in seedlings of single and double T-DNA insertion mutants as well as in silenced amiR lines on vertical ½ MS agar plates. The number of root hairs and average root hair lengths were not significantly different in the mutant and silenced lines when compared to wild-type plantlets (**Supplementary Figure 13**).

In roots, endogenous *ZFP3* transcript levels were comparable to transcripts of *UBIQ10* and *GAPDH2* reference genes, while in leaves, ZFP3 expression was one to two orders of magnitude lower. Estradiol had no significant effect on ZFP3 expression in wild-type plants (**Supplementary Figure 14A**). We were unable to detect *ZFP3* transcripts in root hairs in repeated qRT-PCR experiments (not shown). Histochemical analysis of transgenic plants expressing the pZFP3-GUS marker construct (Joseph et al., 2014) revealed that the *ZFP3* promoter is active in root cells, but is silent or is very weak in root hairs (**Supplementary Figure 14B**). A certain degree of organ-specific differences in *ZFP3* transcript levels was maintained in zfp3amiR silenced lines, which was less striking in ZFP3-overexpressing plants, which had more similar expression in leaves and roots (**Supplementary Figures 15A–C**).

### Analysis of ZFP7-overexpressing plants

ZFP3 belongs to a C2H2 family of ZnF proteins that were suggested to possess partially overlapping functions. To compare functional resemblance or difference of a closely related ZFP factor, transcript profiling of transgenic plants that overexpressed the ZFP7 protein was performed. ZFP7 cDNA was overexpressed in the same pER8-GW expression vector that was used to characterize ZFP3. XVE-ZFP7 plantlets were subjected to the same estradiol treatments that were employed in ZFP3 transcript profiling. Similar to ZFP3 activation, estradiol treatments efficiently enhanced ZFP7 transcript levels in these plants (**Supplementary Figure 16A**). RNAseq analysis revealed that ZFP7 overexpression generated moderate changes in gene expression profiles. A total of 203 genes were upregulated and 296 genes were downregulated by short (6 h) estradiol treatment. Continuous overexpression of ZFP7 by long estradiol treatment led to 10 upregulated and 18 downregulated genes (**Supplementary Figures 16B, C**). These results suggest that fewer target genes are regulated by ZFP7 compared to ZFP3. GO-term analysis revealed that the up- and downregulated gene sets were also different. Among the genes that were upregulated by short ZFP7 overexpression, the following GO categories were enriched: circadian rhythm, response to carbohydrate, cold, salinity, ABA, fungi, and wounding (**Supplementary Figure 17**). GO term categories of genes downregulated by 6 h of ZFP7 overexpression were response to blue light, high light and cold, genes implicated in photosynthesis, circadian rhythm, and post-embryonic development (**Supplementary Figure 18**). Genes related to cell wall formation or root hair development were missing in these categories.

To determine how the ZFP3-repressed genes are regulated by ZFP7 overexpression, RNAseq transcript data of 20 drought and ABA-induced genes and 27 genes implicated in cell wall formation and root hair development were analyzed. Transcription of these genes was not modulated by ZFP7 overexpression in a uniform way, and the tendency for repression or activation could not be identified (**Supplementary Figure 19**, **Supplementary Table 13**).

To verify RNAseq results, transcript levels of six cell wall and root hair-related genes (*RHD6, RSL4, EXPA18, EXT12, RHS12*, and *XTH25*) were analyzed in XVE-ZFP7 plants using RT-qPCR analysis. These genes were downregulated in XVE-ZFP3 plants in an estradiol-dependent manner (**Figure 5C**). Minor variation was found in the transcript levels of the tested genes in XVE-ZFP7 plants, which did not resemble the repression caused by ZFP3 overexpression (**Supplementary Figure 20**).

Although ZFP3 and ZFP7-controlled regulons were different, we decided to test the effect of ZFP7 overexpression on root hair development. The number of root hairs and average root hair lengths were determined on 7-day-old Col-0 and XVE-ZFP7 plantlets, grown on vertical plates using estradiol-containing ½ MS medium. Root hair numbers and root hair lengths were similar in the Col-0 and XVE-ZFP7 plants, suggesting that ZFP7 has no influence on root hair development (**Supplementary Figure 21**).

## Discussion

### ZFP3 do not alter ABA responses in growing plants

ZFP3 is a negative regulator of ABA signaling during germination, and is implicated in photomorphogenesis (Joseph et al., 2014). ABA regulates responses to osmotic stress, promotes seed desiccation, blocks germination, and was shown to inhibit root and leaf growth in young plants (De Smet et al., 2003; Liu et al., 2016; Faragó et al., 2022). In contrast to germination, ZFP3 overexpression did not confer ABA insensitivity to young plants at the postgermination growth phase, but reduced the size of their rosettes and roots, which could be observed with both constitutive and estradiol-inducible ZFP3 gene constructs. Instead of alleviating ABA effect, ZFP3 enhanced growth inhibition in postgermination development. Several genes have been identified that control growth reduction by ABA, such as the HD-ZIP II-type TF ABIG1/HAT22, which restricts shoot growth during water deficiency (Liu et al., 2016). A potassium channel (AKT1), a metabolite transporter (WAT1), and TTG1, which controls flavonoid biosynthesis, were also reported to mediate ABA-controlled growth (Nguyen et al., 2013; Planes et al., 2015). In our transcript profiling experiment, none of these genes were identified to be regulated by ZFP3 (not shown), suggesting that ZFP3 function is independent of these regulators. Rosette and root sizes were similarly reduced by ABA and ZFP3 in *abi4* and *abi5* mutants, suggesting that the ABI4 and ABI5 transcription factors are seed specific (Finkelstein et al., 1998; Finkelstein and Lynch, 2000), and are not implicated in growth control (**Figure 1**, **Supplementary Figure 1**). Our results correlated with earlier reports, showing that inhibition of lateral root growth is independent of ABI4 and ABI5 (De Smet et al., 2003). These results suggest that ZFP3 reduces plant growth independently of ABA-controlled pathways.

Whole genome transcript profiling revealed that ZFP3 overexpression can enhance the transcription of genes implicated in sulfate and glucosinolate metabolism, while many genes in stress and ABA responses, cell wall biogenesis, and modification are downregulated. ZFP3 downregulated the expression of numerous stress-responsive genes in standard growth conditions, but had negligible effect on their ABA activation. Our results suggest that in young plants, overexpression of ZFP3 represses transcription of target genes independently of ABA signals. In contrast to germinating seeds, ZFP3 did not alleviate ABA sensitivity of growing plants, but reduced rosette and root growth further, generating additional inhibition. *ZFP3* silencing had not altered ABA-dependent induction of these genes either (**Figure 4**). Functional redundancy of closely related ZFPs may have compensated the silenced *ZFP3*.

### ZFP3 regulates plant growth and development

GO classification of ZFP3-regulated genes showed that GO terms related to cell wall biosynthesis, modification, cell expansion, and root hair differentiation and elongation were overrepresented among the genes that were downregulated by ZFP3 overexpression. Actually, this was the largest ZFP3-controlled gene category with the highest enrichment (**Supplementary Table 7**, **Supplementary Figure 9**). ZFP3-suppressed genes encode different enzymes and structural or regulatory proteins (**Figure 5**, **Supplementary Table 3**). PMEs modify methyl-esters on pectins, an essential cell wall-associated polysaccharide (Jolie et al., 2010). XTHs mediate the biosynthesis of xyloglucans, which are abundant polysaccharides in primary cell walls, associated with cellulose microfibrils, needed for cell expansion (Liepman et al., 2010). Expansins are conserved cell wall proteins, which are involved in cell wall loosening and are implicated in hormone-dependent cell growth (Li et al., 2002). Hydroxyproline-rich glycoproteins (HRGPs) such as extensins (EXT) are structural components of the plant cell wall. EXTs form a self-assembling protein network that is essential for primary cell wall assembly and cell growth (Lamport et al., 2011). The extracellular leucine-rich repeat extensins (LRXs) are cell wall-anchored signaling proteins, which interact with other proteins through their LRR domain (Draeger et al., 2015). All these proteins are needed for cell wall formation, cell growth, and elongation. ZFP3 overexpression reduced growth and produced aberrant leaves with smaller and less differentiated epidermal cells (**Figures 1**–**3**, **Supplementary Figures 4**-**7**), which can be a consequence of supressed cell wall-related genes. Mutants in such genes often display retarded growth, defects in root hair and pollen elongation, delayed flowering, and reduced fertility (Velasquez et al., 2011; Choudhary et al., 2015; Draeger et al., 2015). A number of ZFP3-repressed cell wall-related genes are also implicated in root hair initiation and elongation, such as *EXPA7* (**Figure 5**, **Supplementary Table 3**). In agreement with our results, RNAi-mediated silencing of *EXPA7* was shown to compromise root hair elongation (Lin et al., 2011). Mutants of EXT and AGP genes have interrupted O-glucosylation and were reported to have irregular root hairs (Velasquez et al., 2011). Besides these examples, a number of other root hair-specific genes were downregulated by ZFP3 overexpression (**Figure 5**, **Supplementary Table 3**). Deficiencies in root hair development and leaf epidermal cell differentiation (**Figures 3**, **7**) can be the consequence of reduced activities of the genes needed for cell wall biogenesis and deposition.

ZFP3 is a member of the C2H2 ZnF protein family. Overexpression of various related ZFP factors were previously shown to confer ABA insensitivity to germinating Arabidopsis seeds (Joseph et al., 2014). ZFP7 was one of those factors that could reduce ABA sensitivity of seed germination. To get information about the molecular function of this factor, ZFP7-regulated gene sets were identified by transcript profiling. RNAseq analysis of ZFP7-overexpressing plants identified hundreds of genes with altered transcript levels. Genome-wide transcript profiling, however, revealed no similarity between ZFP3- and ZFP7-controlled regulons, suggesting that these factors have different functions in growing plants (**Supplementary Figures 16**-**19**, **Supplementary Table 13**). Functional divergence could be confirmed by the difference of their effect on root hair formation. While overexpression of ZFP3 repressed root hair development, ZFP7 had no clear effect on it (**Figure 7**, **Supplementary Figure 21**). These results suggest that despite their similarity, the function of these ZFP proteins can be different.

**Figure 8:**
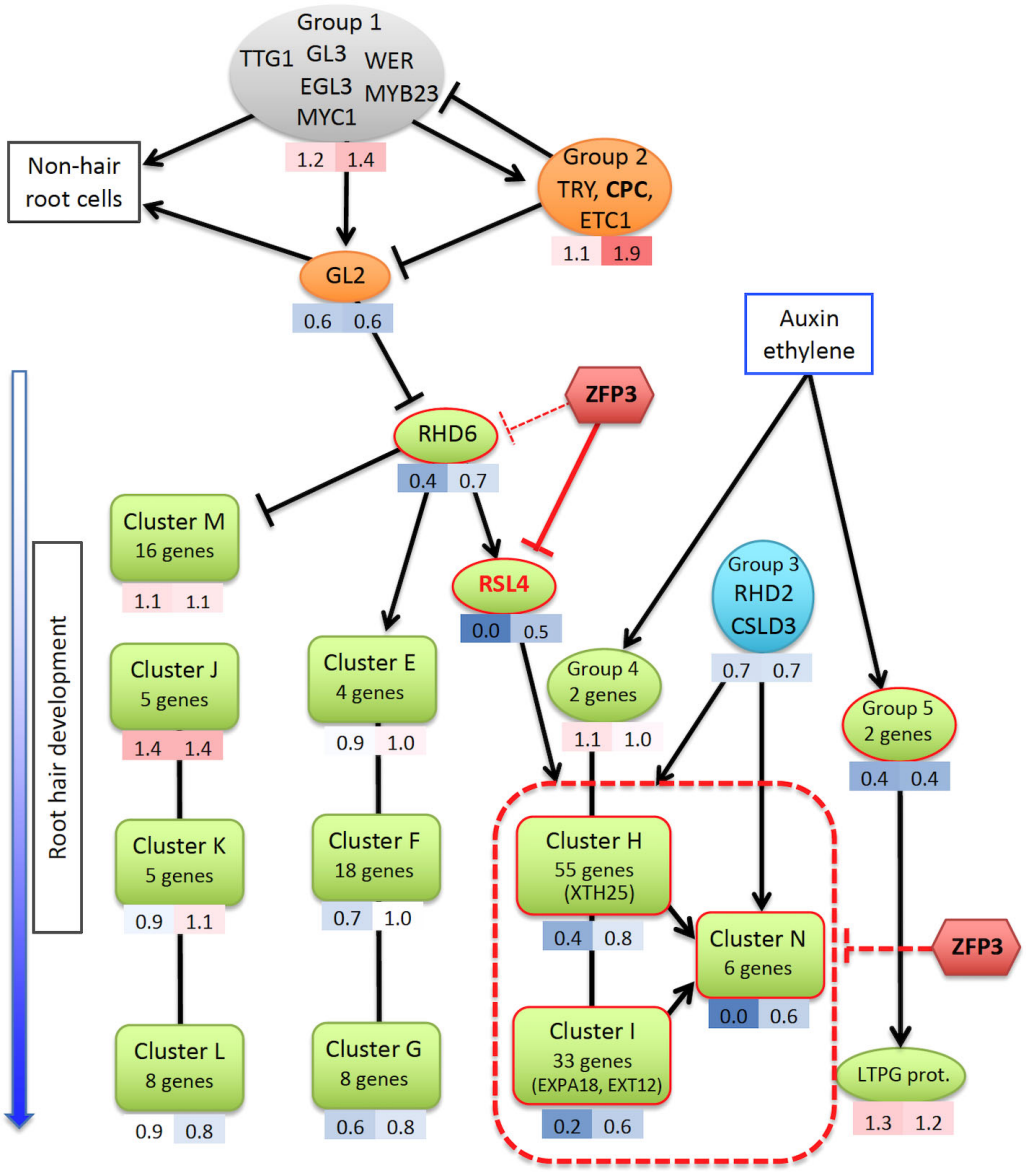
Model of root hair gene network, adapted from Bruex et al. (2012). Transcriptional relationships of core root hair genes are shown, including the early transcription factor complex that determines the fate of root hair and non-hair root cells (gray), the root hair-specific genes (green), other regulatory genes (orange and blue), and ZFP3 (red). Averages of the transcript levels of gene sets (RNAseq) in ZFP3-overexpressing plants are shown under each symbol: left number indicates the effect of short (6 h); right number shows the continuous ZFP3 overexpression (red: induction, blue: repression). Clusters E–N correspond to classification of Bruex et al. (2012). Groups 1–5 indicate sets of genes, which were not included into clusters. Representative genes of several clusters that were studied by qRT-PCR and ChIP are shown in brackets. A list of genes with expression levels of each cluster and group is available in **Supplementary Table 8**.

Root hairs develop from root epidermal cells that are controlled by a well-defined cellular differentiation program. A number of regulatory genes have already been identified that are implicated in root hair initiation or elongation, including transcription and epigenetic factors or protein kinases (Grierson et al, 2014). ZFP3-suppressed genes are involved in root hair morphogenesis, but not in cell-type patterning and hormone action, suggesting that ZFP3 does not interfere with root cell pattern formation, and neither does it have an influence on hormonal regulation (**Supplementary Table 10**) (Grierson et al., 2014). A gene regulatory network with a core collection of 208 genes has been assembled, which determines root epidermal and root hair cell differentiation (Bruex et al., 2012). When transcript levels of those 208 genes were tested in our RNAseq dataset, many root hair-specific regulatory genes were found to be downregulated by ZFP3 (**Supplementary Tables 7**, **8**). Most genes of the hair-specific clusters H, I, and N of the regulatory network model had significantly lower transcript levels after 6 h of ZFP3 overexpression than in wild-type plants, while genes of other clusters were not affected (**Supplementary Table 8**, **Figure 8**) (Bruex et al., 2012). These clusters include genes encoding cell wall proteins (EXPA, EXT, XTH, and PRP), RHS proteins, peroxidases, and signaling factors known to control root hair development (Yi et al., 2010; Bruex et al., 2012; Grierson et al., 2014). Among the most important regulatory genes, *LRX1, IRE, COBL9*, *RSL2*, and *RSL4* were repressed by ZFP3, while *MRH3, CSLD3*, and *RHD2* were not affected (**Figure 5A**, **Supplementary Table 8**). RSL4 is a key bHLH-type transcription factor in root hair initiation and induces a set of genes that are essential for cell wall biogenesis and root hair formation. RSL4 is necessary and sufficient to initiate root hair growth and is a direct target of another bHLH transcription factor, RHD6 (Yi et al., 2010; Datta et al., 2015). There is a considerable overlap between RSL4- and ZFP3-regulated genes as 70% of the RSL4-induced genes were also downregulated by ZFP3 (**Supplementary Table 9**). Binding of ZFP3 to *RSL4* promoter could be demonstrated by ChIP (**Figure 6**), suggesting that this gene is one of the direct targets of ZFP3. It is intriguing that *RSL4* was one of the most repressed genes by ZFP3, suggesting that this gene is a primary target of this ZnF factor. Downregulation of a set of structural genes belonging to Clusters H, I, and N in the Bruex model can be a consequence of silenced RSL4 (**Figure 8**). Our data suggest that ZFP3 can interfere with the transcription regulatory network that controls root hair development at least partially through inhibition of *RSL4*. Besides ZFP3, other ZnF proteins were shown to modulate root hair morphology in various ways. ZFP5 functions as a positive regulator of root hair initiation by directly promoting the expression of the R3-type MYB factor CAPRICE (CPC) (An et al., 2012). ZFP5 and ZFP6 are closely related and promote trichome formation by integrating hormonal signals (Zhou et al., 2013). Recently, ZFP5 was found to promote root hair development in response to phosphate or potassium starvation, and mediate ethylene signals in activation of key regulatory genes such as *RSL4* (Huang et al., 2020). Another ZnF, AtZP1, was recently reported to inhibit root hair development by acting as a repressor of *RHD6*, *RSL2*, and *RSL4*, downstream of *GL2* (Han et al., 2020). Many genes that were downregulated by AtZP1 were also repressed by ZFP3, and their action seems to converge on *RSL2* and *RSL4* in the transcription network. ZFP3 seems to function as a repressor in root hair development by interfering with the transcription regulatory network through inhibition of the activity of key regulatory genes such as *RSL4*, which is needed to activate sets of structural genes encoding cell wall proteins (**Figure 8**).

## Data availability statement

The datasets presented in this study can be found in online repositories. The names of the repository/repositories and accession number(s) can be found below: https://www.ncbi.nlm.nih.gov/, PRJNA731325 https://www.ncbi.nlm.nih.gov/, PRJNA1029453.

## Author contributions

DB Performed most experiments, EB analysed RNAseq data, DF analysed phenotyping data, GR made gene cloning, plant phenotyping, ID, NL made electron microscopy, LZ. analysed data, MP analysed NGS datasets, IN made RNAseq NGS sequencing, LS. directed research, analysed data and wrote the manuscript. All authors contributed to the article and approved the submitted version.
