# Supplementary material for: The zinc finger protein 3 of Arabidopsis thaliana regulates vegetative growth and root hair development
Source: Front Plant Sci. 2024 Jan 5;14:1221519. doi: 10.3389/fpls.2023.1221519 (PMC10796524; doi:10.3389/fpls.2023.1221519)
Supplement: Supplementary file 1 [file DataSheet_1.pdf]

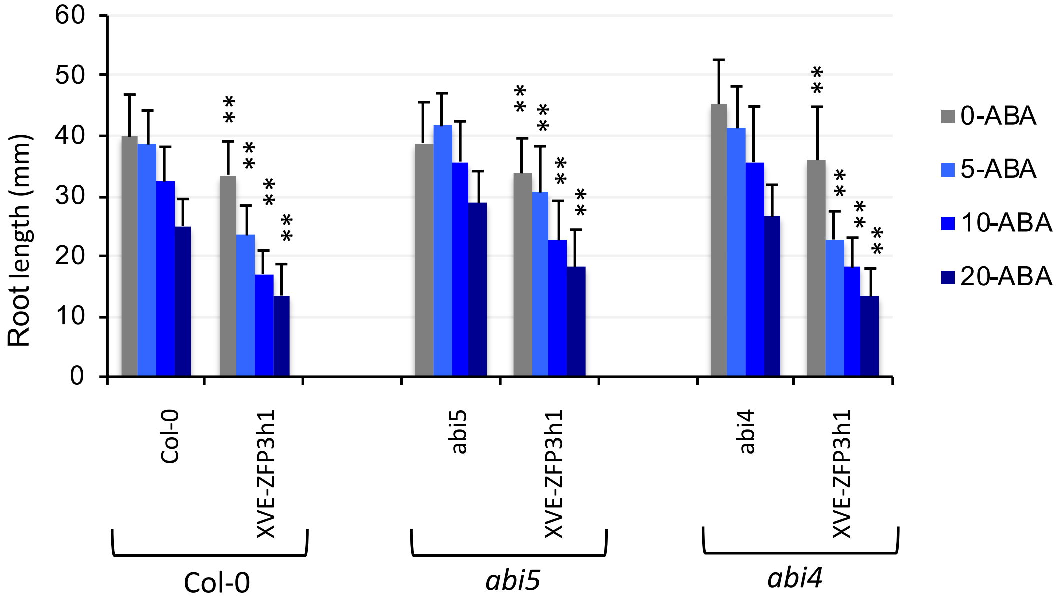

Figure S1. Root growth of ZFP3 overexpressing plants in Col-0 wild type, *abi5* and *abi4* mutant background. 5 days-old seedlings were transferred to culture media supplemented by 5 μM estradiol and 0, 5, 10 or 20 μM ABA and plantlets were cultured on vertical plates. Root lengths were measured after 7 days of growth. Error bars indicate standard deviation (N=35). Significant differences between root lenghts of ZFP3 overexpressing plants and their respective backgrounds are shown by * ($p<0.05$) and ** ($p<0.01$) (Ttest).

A

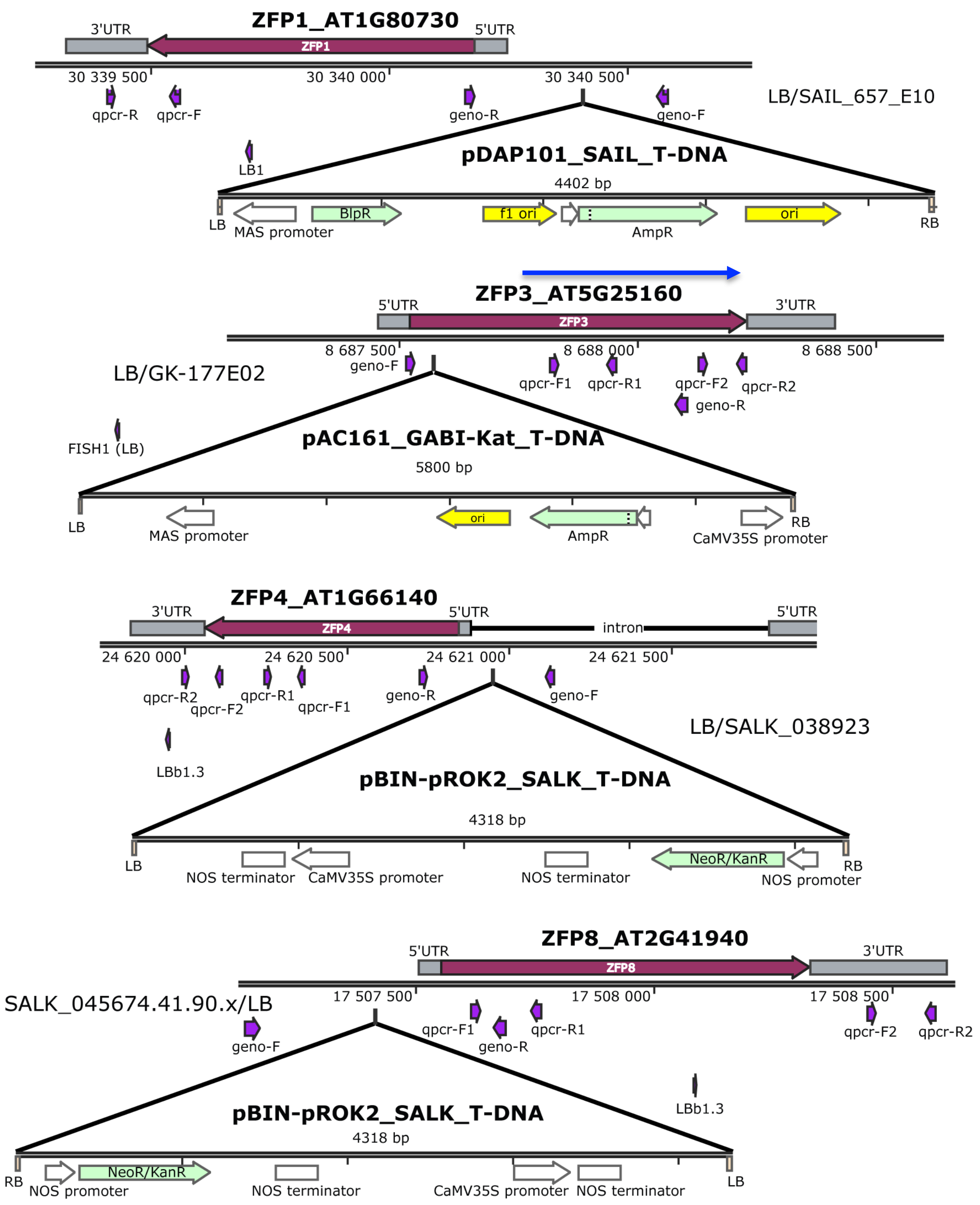

B

Mutant	Gene	Rel.expr.	SD
SAIL_657_E10	ZFP1	7.09	1.48
GK-177E02	ZFP3	0.61	0.13
SALK_038923	ZFP4	0.94	0.07
SALK_045674	ZFP8	0.89	0.14

Figure S2. Positions of T-DNA insertions disrupting *ZFP1*, *ZFP3*, *ZFP4* and *ZFP7* genes. A) Schematic map of the ZFP genes, indicating the positions of T-DNA insertions, the T-DNA-encoded genes and the primers used for genotyping and qRT-PCR analysis. A blue arrow in the *ZFP3* gene indicates the position of the predicted short ORF which may encode a truncated protein. B) Relative transcript levels of the genes in the homozygous mutants. 1 corresponds to the transcript of Col-0 wild type plants.

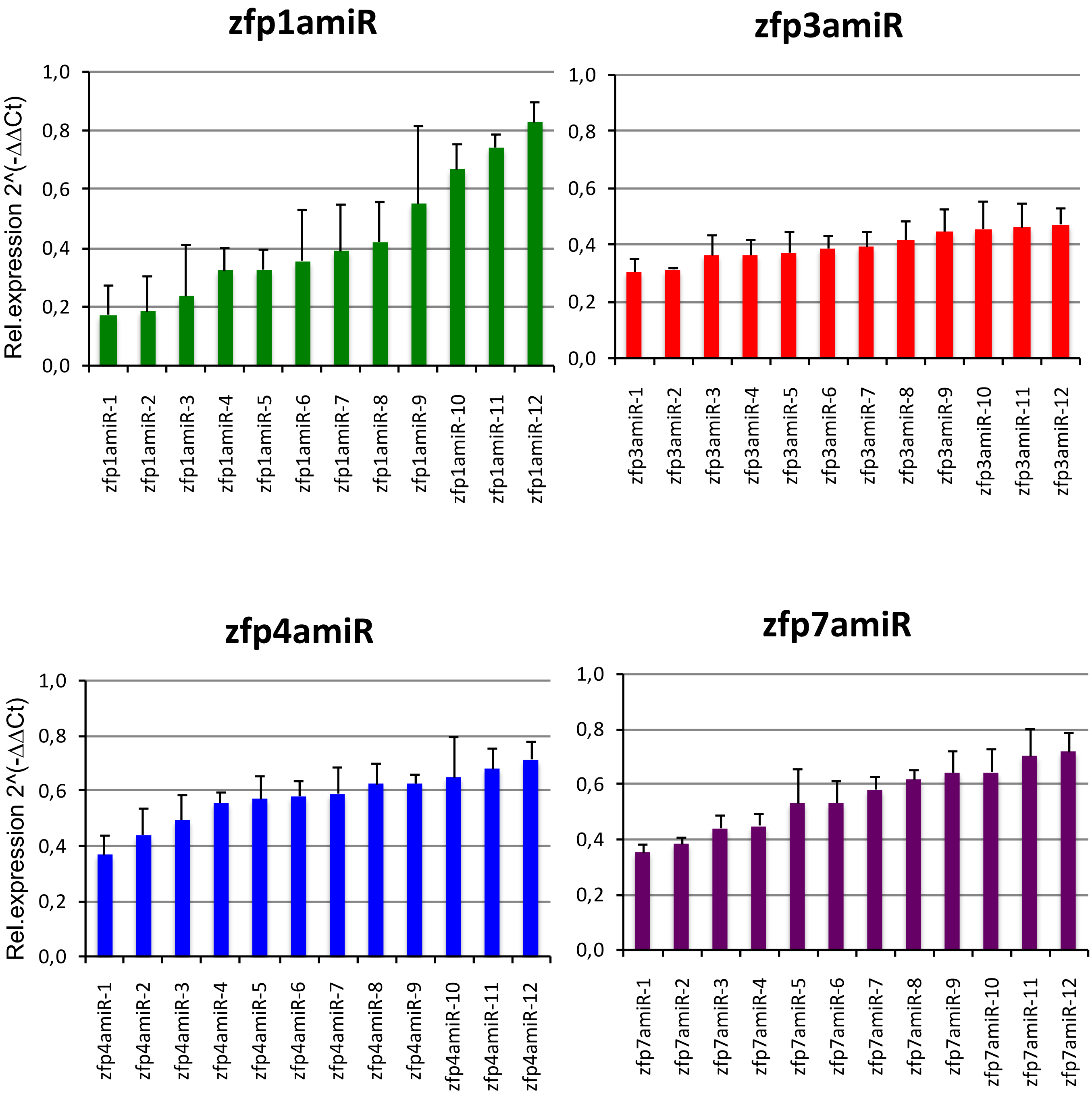

Figure S3. Expression of *ZFP1*, *ZFP3*, *ZFP4* and *ZFP7* genes in silenced Arabidopsis lines. ZFP genes were repressed with artificial microRNA constructs controlled by the CaMV35S promoter. Relative transcript levels of 12 independent lines for each constructs are shown, where 1 corresponds to expression in Col-0 wild type plants. The lines with most efficient silencing were used for further analysis.

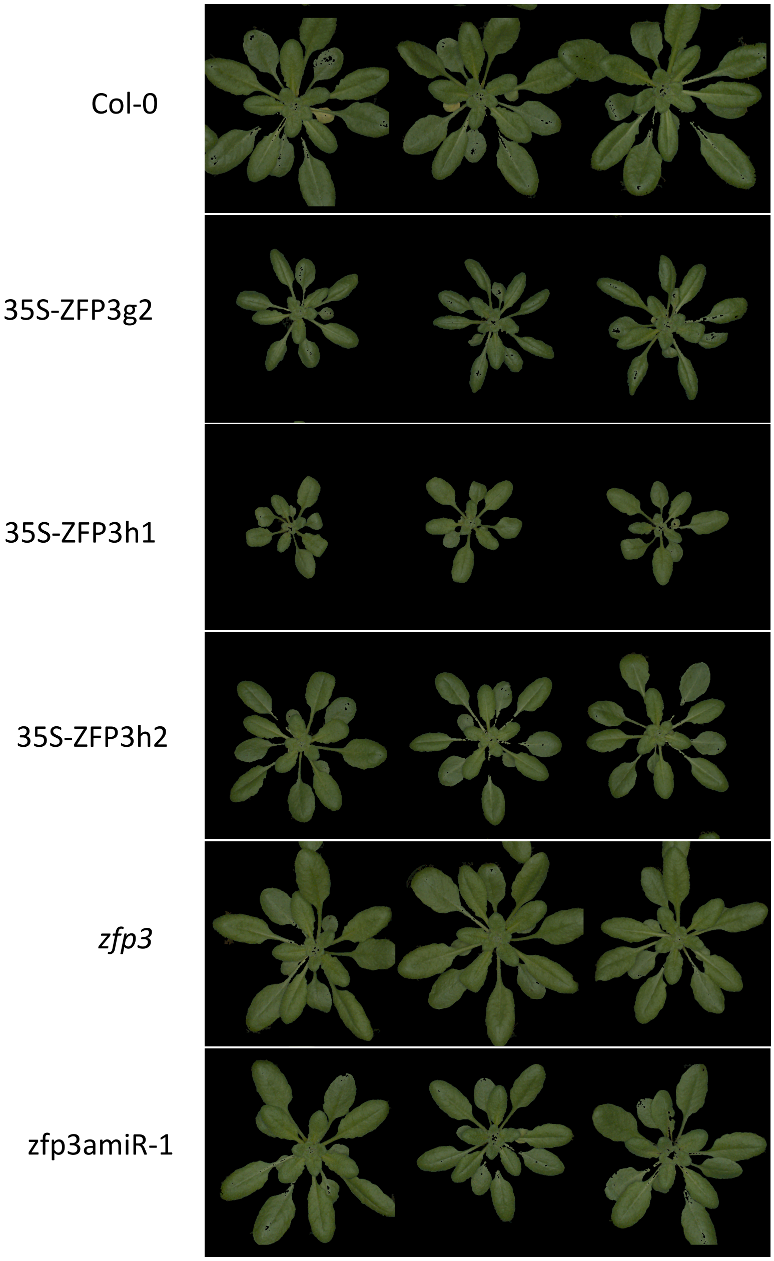

Figure S4. Rosette images of Arabidopsis plants grown in phenotypic platform for 30 days. RGB images were masked to remove the background. Typical plants from Col-0 wild type, ZFP3 overexpressing lines (35S-ZFP3g2, 35S-ZFP3h1, 35S-ZFP3h2), *zfp3* T-DNA insertion mutant and the zfp3amiR-1 silenced line are shown.

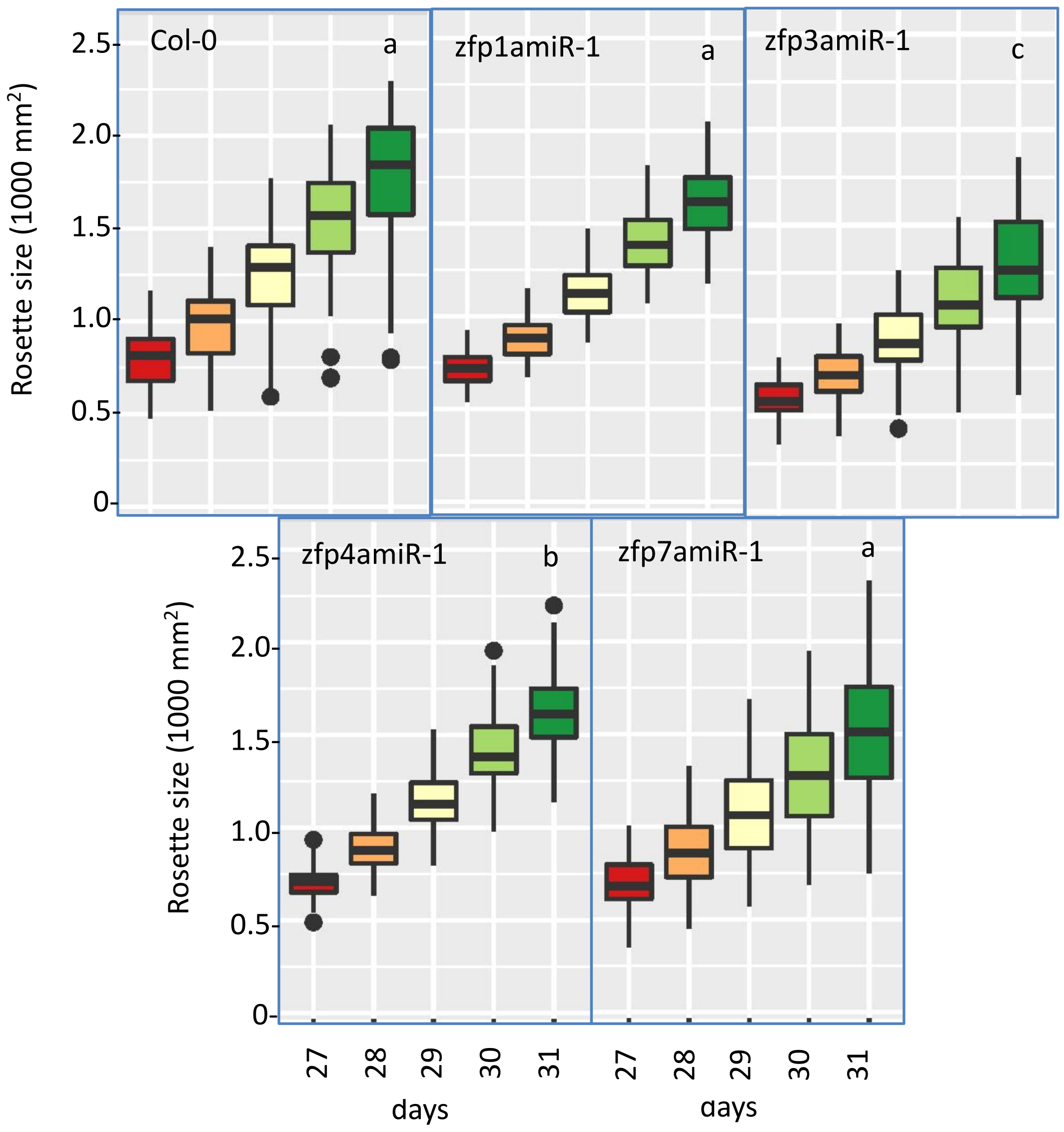

Figure S5. Rosette sizes of transgenic plants with silenced *ZFP1, ZFP3, ZFP4* and *ZFP7* genes. Plants were grown in a plant phenotyping platform (PSI) and rosette sizes were photographed between day 27 to 31 at daily intervals. Analysis of variance was performed (Kruskal-Wallis) with pairwise Wilcoxon test / Mann-Whitney test of significance. Different letters indicate significant differences (N=40, p-value < 0.05).

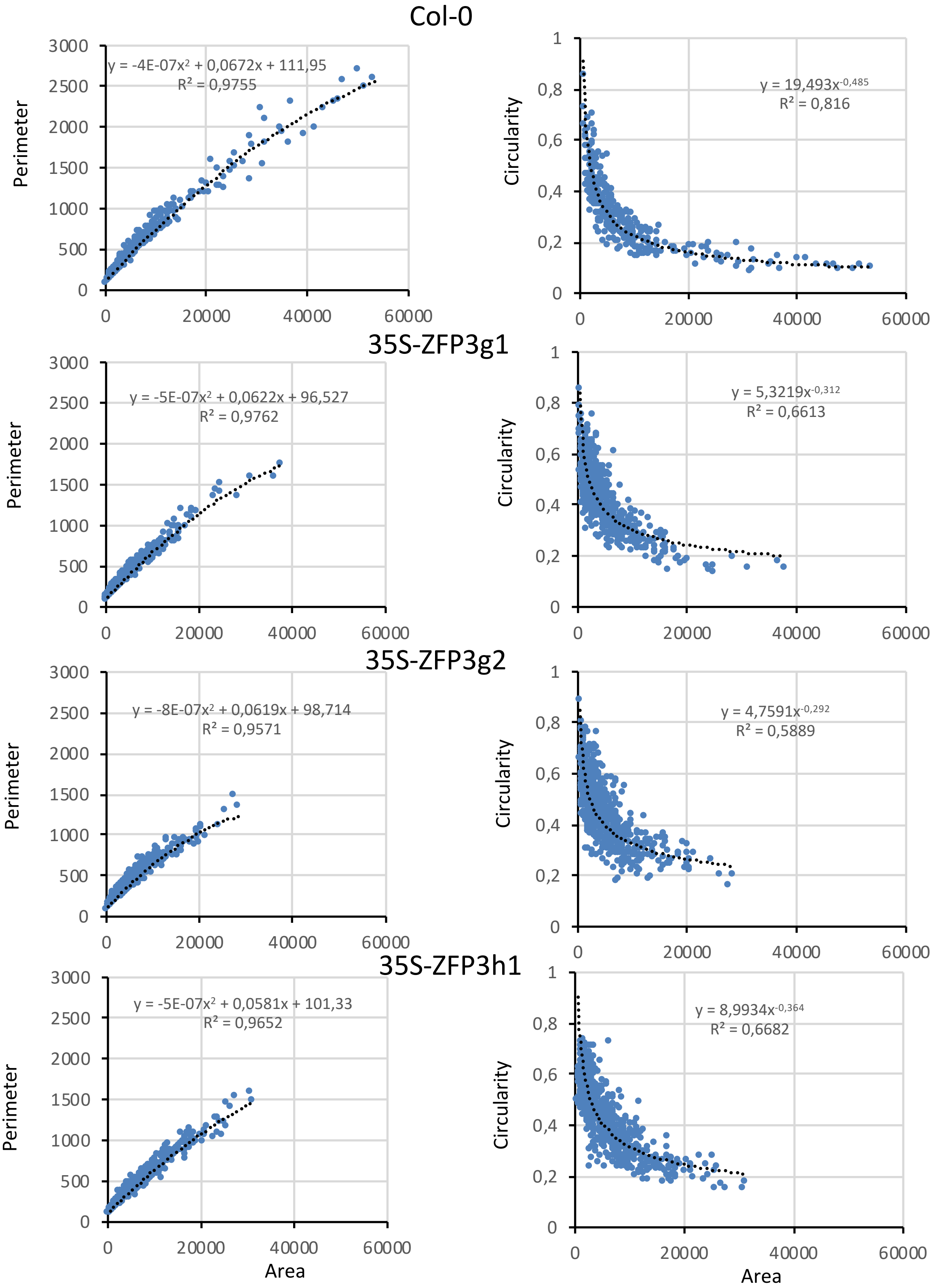

Figure S6. Cell size and shape distribution of epidermal cells on ***adaxial*** leaf surface of wild type (Col-0) and ZFP3 overexpressing plants. Images were taken by scanning electron microscopy as shown on Figure 3A, and cell sizes were measured with modified PlantSize software (Faragó et al., 2018, *Front Plant Sci* **9**: 219). Plots of Perimeter (pixels) and Area (pixels) and Circularity and Area are shown, each dot represent a single cell. Note that Area and Perimeter correlate positively, while Circularity has a reverse correlation with Area.

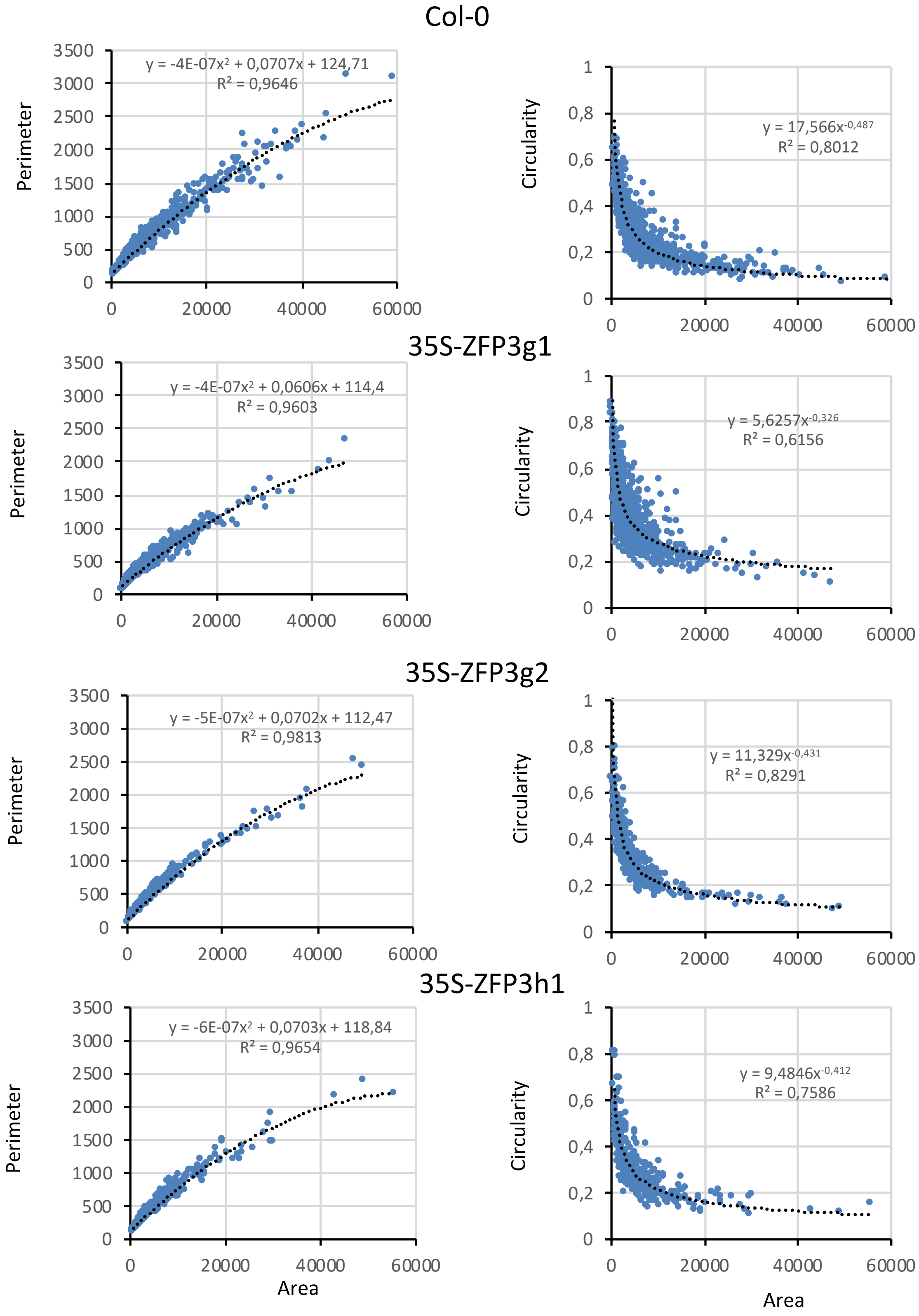

Figure S7. Cell size and shape distribution of epidermal cells on ***abaxial*** leaf surface of wild type (Col-0) and ZFP3 overexpressing plants. Images were taken by scanning electron microscopy as shown on Figure 3A, and cell sizes were measured with modified PlantSize software (Faragó et al., 2018, *Front Plant Sci* **9**: 219). Plots of Perimeter (pixels) and Area (pixels) and Circularity and Area are shown, each dot represent a single cell. Note that Area and Perimeter correlate positively, while Circularity has a reverse correlation with Area.

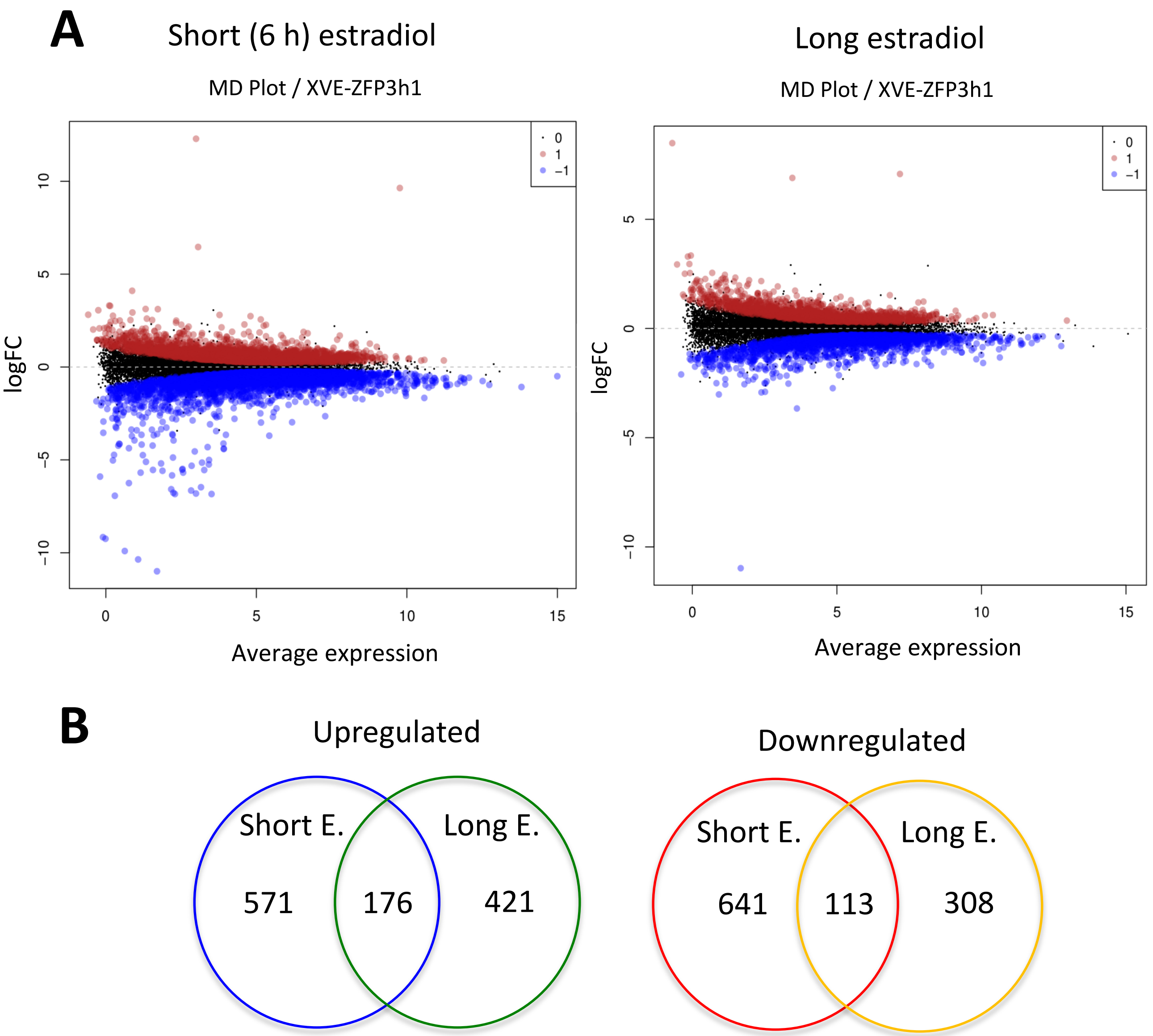

Figure S8.
Summary of the ZFP3 transcript profiling experiment. (A) MD plot of misregulated gene sets from short (6 h) and long (continuous) estradiol-treated plants. Mean Difference plot indicates the log2(Fold Change) values versus mean logCPM expression values, highlighted genes have FDR<0.05. + and - values indicate up and down regulation, respectively. (B) Number of up and downregulated genes (at least 2.5 fold change) in ZFP3 overexpressing plants. Short E. and Long E. indicates 6 h or continuous estradiol treatment of XVE-ZFP3h1 plants.

GO Molecular Function

GO:MF		stats	
Term name	Term ID	p_{adj}	$-\log_{10}(p_{adj})$
heme binding	GO:0020037	3.105×10^{-13}	
tetrapyrrole binding	GO:0046906	1.277×10^{-12}	
structural constituent of cell wall	GO:0005199	1.224×10^{-9}	
oxidoreductase activity, acting on peroxide as acceptor	GO:0016684	1.447×10^{-8}	
peroxidase activity	GO:0004601	4.909×10^{-8}	
oxidoreductase activity	GO:0016491	3.144×10^{-7}	
monooxygenase activity	GO:0004497	5.333×10^{-7}	
antioxidant activity	GO:0016209	1.226×10^{-6}	
oxidoreductase activity, acting on paired donors, with in...	GO:0016705	1.210×10^{-5}	
oxidoreductase activity, acting on paired donors, with in...	GO:0016709	8.828×10^{-5}	
xyloglucan:xyloglucosyl transferase activity	GO:0016762	3.468×10^{-3}	
iron ion binding	GO:0005506	1.175×10^{-2}	
oxidoreductase activity, acting on metal ions	GO:0016722	1.462×10^{-2}	
metal ion binding	GO:0046872	2.730×10^{-2}	
cation binding	GO:0043169	3.183×10^{-2}	

1 to 1[illegible]

GO Biological Process

GO:BP		stats	
Term name	Term ID	p_{adj}	$-\log_{10}(p_{adj})$
cell wall organization	GO:0071555	4.301×10^{-13}	
external encapsulating structure organization	GO:0045229	5.191×10^{-12}	
cell wall organization or biogenesis	GO:0071554	1.790×10^{-10}	
hydrogen peroxide catabolic process	GO:0042744	2.740×10^{-9}	
plant-type cell wall organization	GO:0009664	7.259×10^{-8}	
hydrogen peroxide metabolic process	GO:0042743	9.962×10^{-8}	
plant-type cell wall organization or biogenesis	GO:0071669	2.332×10^{-7}	
trichoblast differentiation	GO:0010054	2.739×10^{-7}	
obsolete oxidation-reduction process	GO:0055114	6.686×10^{-7}	
root epidermal cell differentiation	GO:0010053	2.276×10^{-6}	
trichoblast maturation	GO:0048764	6.770×10^{-6}	
root hair cell differentiation	GO:0048765	6.770×10^{-6}	
reactive oxygen species metabolic process	GO:0072593	7.607×10^{-6}	
cell maturation	GO:0048469	7.792×10^{-6}	
root development	GO:0048364	1.001×10^{-5}	
root system development	GO:0022622	1.111×10^{-5}	
plant epidermal cell differentiation	GO:0090627	1.229×10^{-5}	
root morphogenesis	GO:0010015	2.157×10^{-5}	
plant epidermis development	GO:0090558	3.149×10^{-5}	
anatomical structure maturation	GO:0071695	5.165×10^{-5}	
developmental maturation	GO:0021700	2.332×10^{-4}	
cellular oxidant detoxification	GO:0098869	1.012×10^{-3}	
cellular detoxification	GO:1990748	1.350×10^{-3}	
cellular response to toxic substance	GO:0097237	1.483×10^{-3}	
cell-cell junction assembly	GO:0007043	1.666×10^{-3}	
plant organ development	GO:0099402	3.522×10^{-3}	
cell junction assembly	GO:0034329	4.899×10^{-3}	
root hair cell development	GO:0080147	7.737×10^{-3}	
isoprenoid metabolic process	GO:0006720	9.164×10^{-3}	
xyloglucan metabolic process	GO:0010411	9.847×10^{-3}	
cell wall biogenesis	GO:0042546	1.436×10^{-2}	
cell development	GO:0048468	1.521×10^{-2}	
response to oxidative stress	GO:0006979	1.989×10^{-2}	
detoxification	GO:0098754	2.515×10^{-2}	
response to acid chemical	GO:0001101	3.163×10^{-2}	
cell-cell junction organization	GO:0045216	3.874×10^{-2}	
plant organ morphogenesis	GO:1905392	4.030×10^{-2}	

1 to 3[illegible]

GO Cellular Component

GO:CC		stats	
Term name	Term ID	p_{adj}	$-\log_{10}(p_{adj})$
external encapsulating structure	GO:0030312	1.272×10^{-12}	
cell wall	GO:0005618	7.029×10^{-12}	
extracellular region	GO:0005576	2.847×10^{-9}	
cell periphery	GO:0071944	1.301×10^{-8}	
plant-type cell wall	GO:0009505	3.154×10^{-6}	
intrinsic component of membrane	GO:0031224	1.283×10^{-4}	
integral component of membrane	GO:0016021	1.647×10^{-4}	
apoplast	GO:0048046	8.161×10^{-3}	
Casparian strip	GO:0048226	8.296×10^{-3}	
secondary cell wall	GO:0009531	4.934×10^{-2}	

1 to 1[illegible]

Figure S9 .
GO term categories of genes downregulated by 6 hours of ZFP3 overexpression (short treatment). Data obtained with g:Profiler: https://biit.cs.ut.ee/gprofiler/gost.

GO KEGG categories

KEGG		stats	
Term name	Term ID	p_{adj}	$-\log_{10}(p_{adj})$
Phenylpropanoid biosynthesis	KEGG:00940	1.997×10^{-12}	
Biosynthesis of secondary metabolites	KEGG:01110	1.016×10^{-6}	
Metabolic pathways	KEGG:01100	2.003×10^{-2}	
Pentose and glucuronate interconversions	KEGG:00040	2.886×10^{-2}	
Terpenoid backbone biosynthesis	KEGG:00900	3.167×10^{-2}	

1 to [illegible]

GO Molecular Function

GO:MF		stats	
Term name	Term ID	p_{adj}	$-\log_{10}(p_{adj})$
glycerol-3-phosphate O-acyltransferase activity	GO:0004366	2.802×10^{-2}	
sn-1-glycerol-3-phosphate C16:0-DCA-CoA acyl transf...	GO:0102420	2.802×10^{-2}	
1-aminocyclopropane-1-carboxylate synthase activity	GO:0016847	2.802×10^{-2}	
glycerol-3-phosphate 2-O-acyltransferase activity	GO:0090447	4.170×10^{-2}	

GO Biological Process

GO:BP		stats	
Term name	Term ID	p_{adj}	$-\log_{10}(p_{adj})$
aging	GO:0007568	4.600×10^{-5}	
response to stimulus	GO:0050896	1.484×10^{-3}	
fruit ripening	GO:0009835	1.976×10^{-3}	
plant organ senescence	GO:0090693	6.284×10^{-3}	
secondary metabolic process	GO:0019748	1.318×10^{-2}	
suberin biosynthetic process	GO:0010345	3.377×10^{-2}	
leaf senescence	GO:0010150	3.481×10^{-2}	

GO Cellular Component

GO:CC		stats	
Term name	Term ID	p_{adj}	$-\log_{10}(p_{adj})$
external encapsulating structure	GO:0030312	6.491×10^{-8}	
cell wall	GO:0005618	1.733×10^{-7}	
extracellular region	GO:0005576	2.482×10^{-5}	
cell periphery	GO:0071944	4.973×10^{-5}	
plant-type cell wall	GO:0009505	1.689×10^{-2}	

GO KEGG categories

KEGG		stats	
Term name	Term ID	p_{adj}	$-\log_{10}(p_{adj})$
Metabolic pathways	KEGG:01100	7.637×10^{-3}	
Glycerolipid metabolism	KEGG:00561	2.571×10^{-2}	

MIRNA		stats	
Term name	Term ID	p_{adj}	$-\log_{10}(p_{adj})$
ath-miR857	MIRNA:ath-mi...	4.998×10^{-2}	

Figure S10. GO term categories of genes downregulated by continuous ZFP3 overexpression (long treatment). Data obtained with g:Profiler: https://biit.cs.ut.ee/gprofiler/gost

GO Molecular Function

GO:MF		stats	
Term name	Term ID	p_{adj}	$-\log_{10}(p_{adj})$
glutathione transferase activity	GO:0004364	1.843×10^{-3}	
DNA-binding transcription factor activity	GO:0003700	3.209×10^{-2}	

1 to

GO Biological Process

GO:BP		stats	
Term name	Term ID	p_{adj}	$-\log_{10}(p_{adj})$
glucosinolate biosynthetic process	GO:0019761	4.637×10^{-11}	
glycosinolate biosynthetic process	GO:0019758	4.637×10^{-11}	
S-glycoside biosynthetic process	GO:0016144	4.637×10^{-11}	
sulfur compound metabolic process	GO:0006790	9.411×10^{-11}	
sulfur compound biosynthetic process	GO:0044272	3.495×10^{-10}	
glycosyl compound biosynthetic process	GO:1901659	1.118×10^{-9}	
S-glycoside metabolic process	GO:0016143	9.523×10^{-8}	
glycosinolate metabolic process	GO:0019757	9.523×10^{-8}	
glucosinolate metabolic process	GO:0019760	9.523×10^{-8}	
secondary metabolic process	GO:0019748	3.043×10^{-7}	
glycosyl compound metabolic process	GO:1901657	8.438×10^{-7}	
secondary metabolite biosynthetic process	GO:0044550	2.799×10^{-6}	
cellular response to sulfur starvation	GO:0010438	2.532×10^{-5}	
toxin metabolic process	GO:0009404	4.105×10^{-4}	
response to insect	GO:0009625	8.695×10^{-3}	
toxin catabolic process	GO:0009407	8.939×10^{-3}	
chromosome organization involved in meiotic cell cycle	GO:0070192	1.204×10^{-2}	
homologous chromosome segregation	GO:0045143	1.554×10^{-2}	
detoxification	GO:0098754	2.264×10^{-2}	
meiotic chromosome segregation	GO:0045132	4.909×10^{-2}	

1 to 20

GO Cellular Component

GO:CC		stats	
Term name	Term ID	p_{adj}	$-\log_{10}(p_{adj})$
condensed chromosome	GO:0000793	1.385×10^{-2}	

1 to

GO KEGG categories

KEGG		stats	
Term name	Term ID	p_{adj}	$-\log_{10}(p_{adj})$
Glucosinolate biosynthesis	KEGG:00966	7.409×10^{-11}	
2-Oxocarboxylic acid metabolism	KEGG:01210	5.474×10^{-6}	
Sulfur metabolism	KEGG:00920	1.527×10^{-2}	
Glutathione metabolism	KEGG:00480	3.580×10^{-2}	

1 to

WP		stats	
Term name	Term ID	p_{adj}	$-\log_{10}(p_{adj})$
Glucosinolate biosynthesis (from methionine)	WP:WP4597	1.980×10^{-8}	
Glucosinolate biosynthesis (from aromatic amino acid)	WP:WP4598	6.276×10^{-3}	

1 to

Figure S11. GO term categories of genes upregulated by 6 hours of ZFP3 overexpression (short treatment). Data obtained with g:Profiler (https://biit.cs.ut.ee/gprofiler/gost).

GO Molecular Function

GO:MF		stats	
Term name	Term ID	p_{adj}	$-\log_{10}(p_{adj})$
6-methylthiopropyl glucosinolate S-oxygenase activity	GO:0080105	4.984×10^{-2}	

GO Biological Process

GO:BP		stats	
Term name	Term ID	p_{adj}	$-\log_{10}(p_{adj})$
stomatal complex development	GO:0010374	1.983×10^{-3}	

GO KEGG categories

KEGG		stats	
Term name	Term ID	p_{adj}	$-\log_{10}(p_{adj})$
Starch and sucrose metabolism	KEGG:00500	2.624×10^{-2}	

WP		stats	
Term name	Term ID	p_{adj}	$-\log_{10}(p_{adj})$
Flavonoid Biosynthesis	WP:WP1538	4.985×10^{-2}	

Figure S12. GO term categories of genes upregulated by continuous ZFP3 overexpression (long treatment). Data obtained with g:Profiler: https://biit.cs.ut.ee/gprofiler/gost.

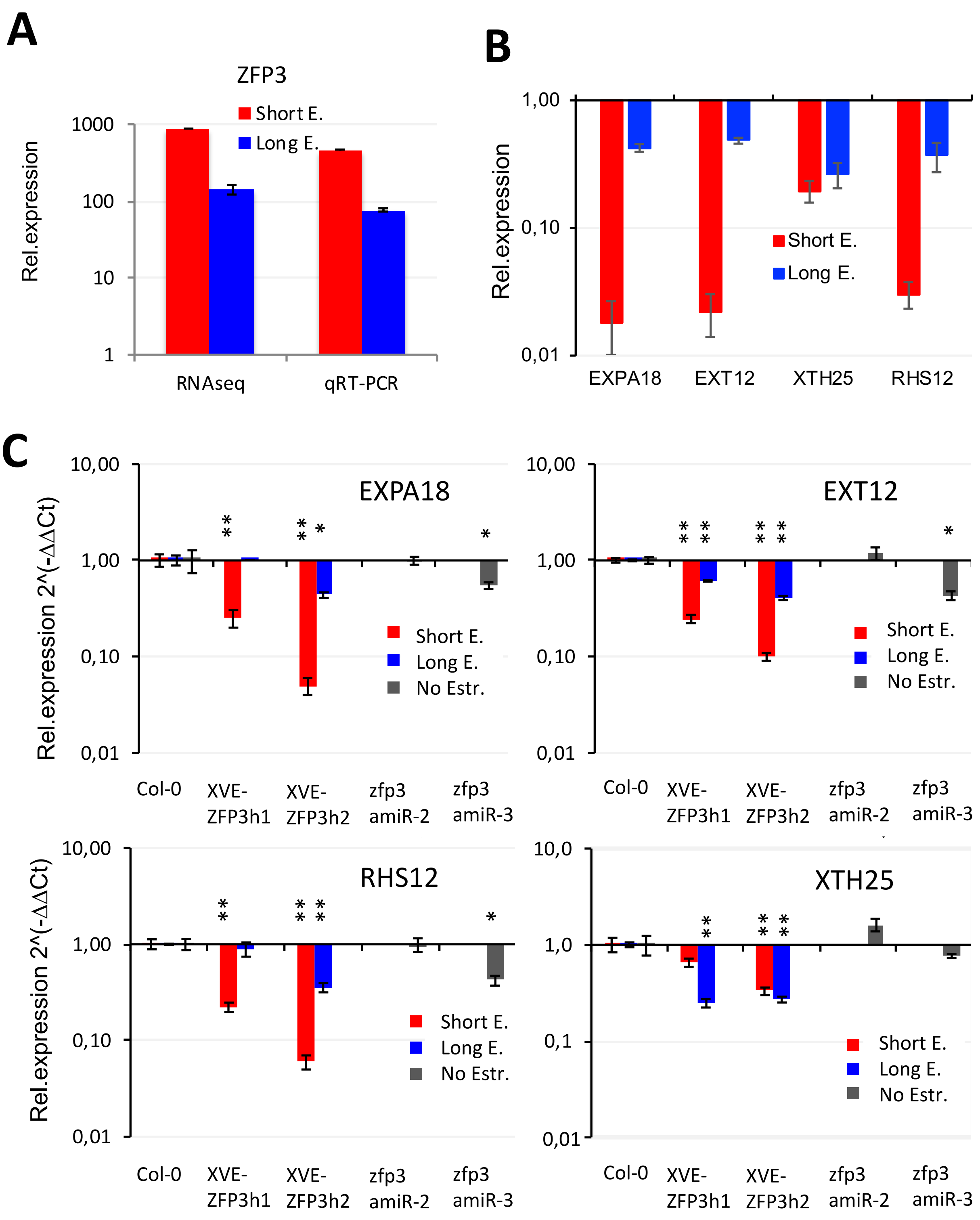

Figure S13. Verification of RNAseq transcript profiling data in independent expression analysis. a) Transcript levels of ZFP3 in XVE-ZFP3h1 plants after short and long estradiol treatment, with detection of RNAseq and qRT-PCR method. b) Relative transcript levels of four cell wall genes determined in RNAseq experiment. Expression is show in log scale, where 1 corresponds to wild type plants (=1). c) qRT-PCR analysis of the four cell wall genes. Transcript levels were determined in two independent XVE-ZFP3h and zfp3amiR lines. Error bars indicate standard deviation (N=3), significant differences between transcript levels of Col-0 and transgenic plants are shown by * ($p<0.05$) and ** ($p<0.01$) (Ttest).

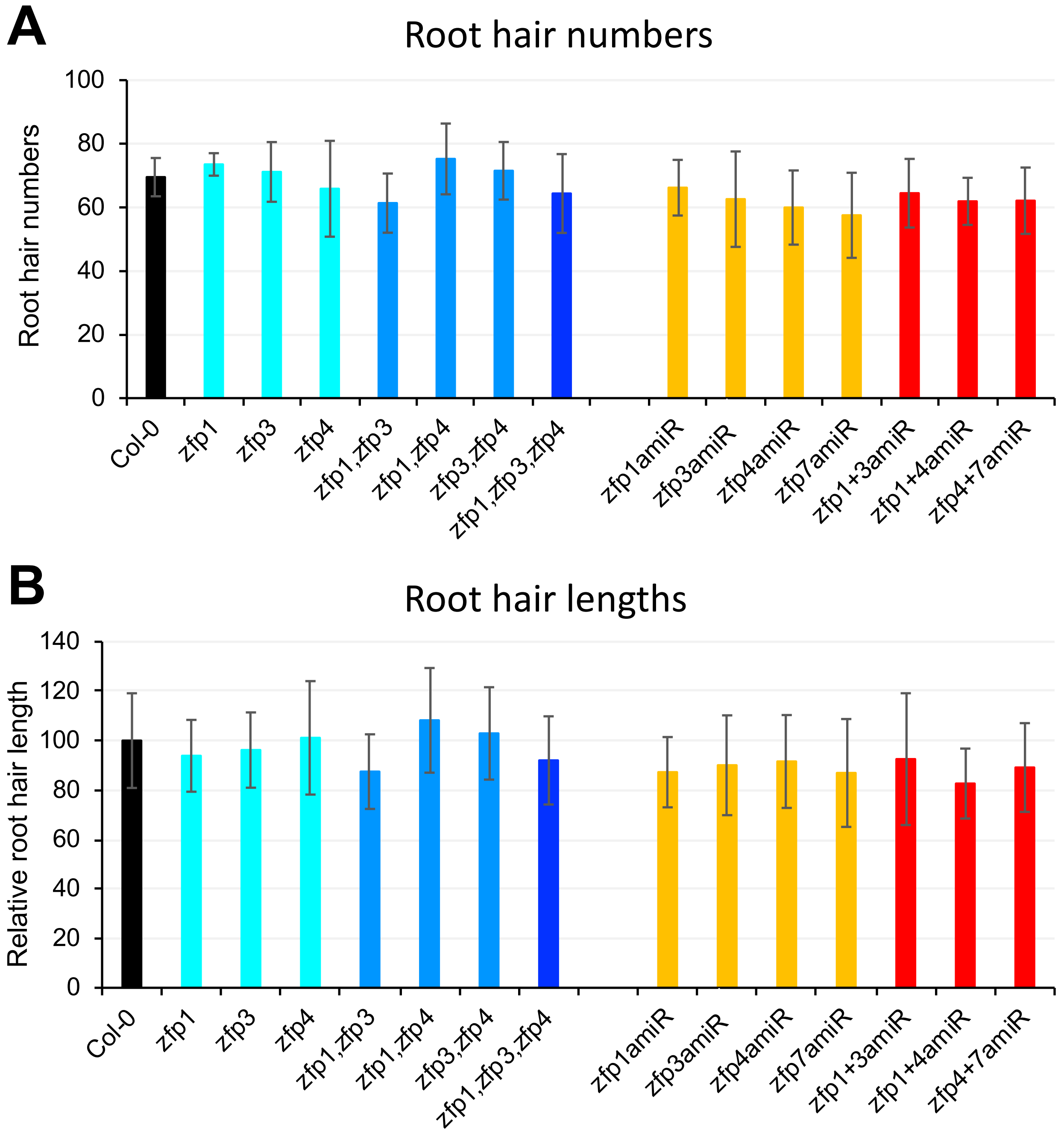

Figure S14. Root hair formation of Arabidopsis *zfp1, zfp3* and *zfp4* single, *zfp1zfp3, zfp1zfp4 and zfp3zfp4* double and *zfp1zfp3zfp4* triple mutants and Arabidopsis lines with silenced *ZFP1, ZFP2, ZFP4* and *ZFP7* genes and combination of amiR silenced *ZFP1,ZFP3, ZFP1,ZFP4* and *ZFP4,ZFP7* genes. Seeds were germinated and seedlings cultured on vertical ½ MS media agar plates. Roots were photographed on 7 days-old seedlings. A) Number of root hairs on 7 days-old seedlings. B) Average lengths of root hairs on 7 days-old seedlings. Root hair lengths were measured with the ImageJ software and normalized to average of wild type plants. Error bars on diagrams indicate standard error.

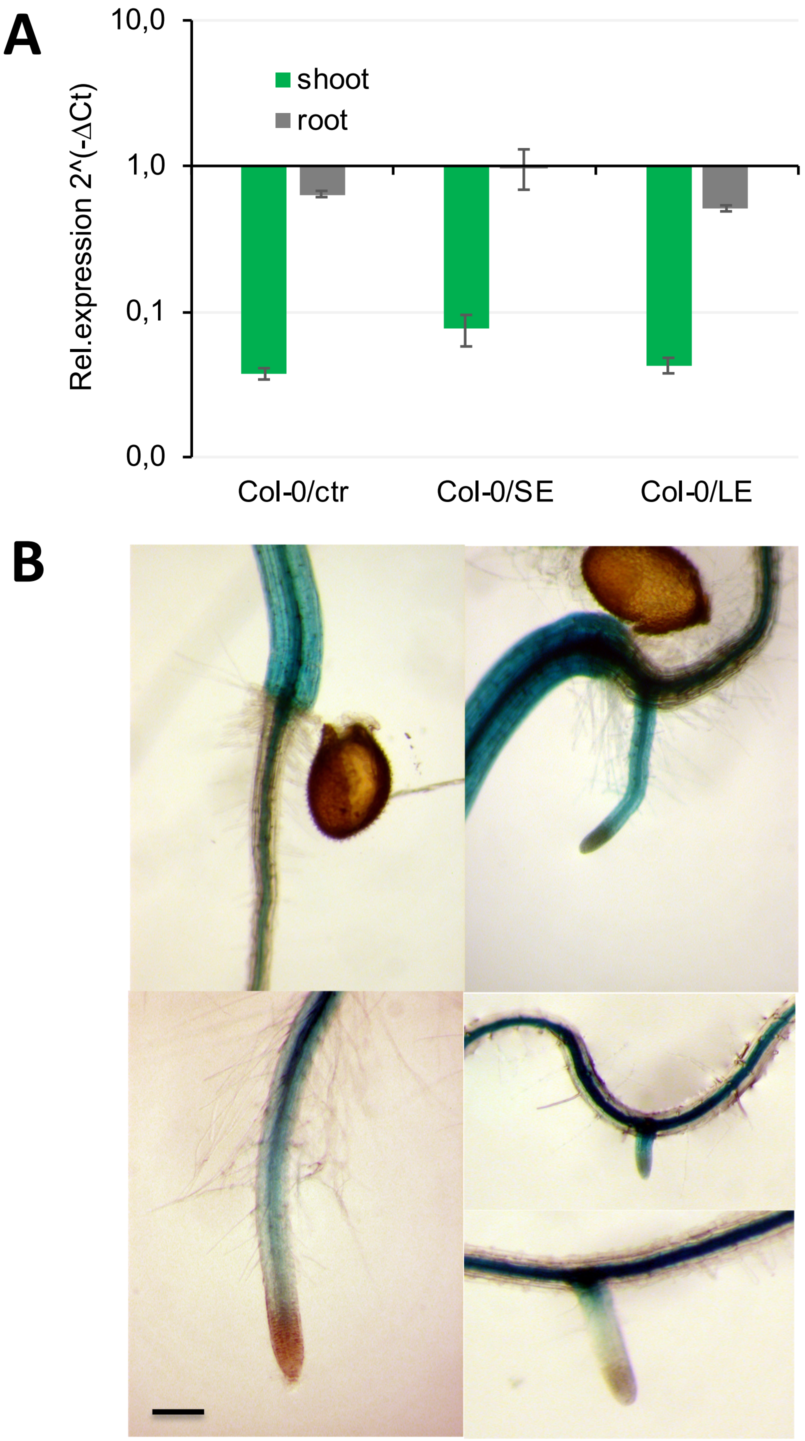

Figure S15. Expression of ZFP3 in wild type Arabidopsis plants. A) Transcript levels of ZFP3 gene in shoots and roots of Col-0 wild type plants subjected to short and long estradiol treatments (SE and LE, respectively). Relative transcript levels are shown in logarithmic scale using reference genes UBIQ10 and GAPDH2. B) pZFP3-GUS activity in roots and root hairs. Histochemical staining was made on roots of pZFP3-GUS expressing plantlets (Joseph et al., 2014, *Plant Physiol* **165**(3): 1203-1220). Scale bar = 200 μm.

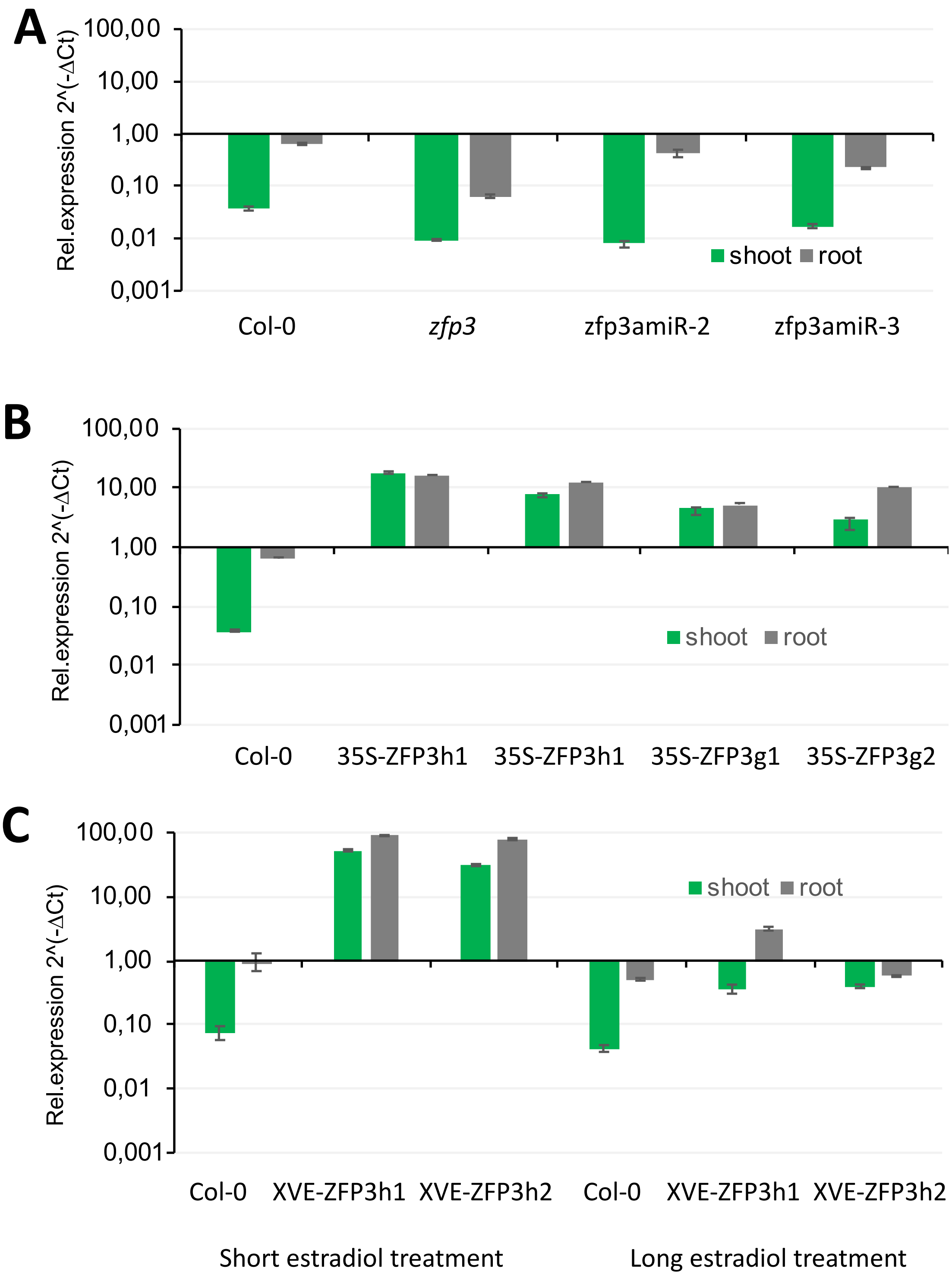

Figure S16. Transcript analysis of *ZFP3* in Arabidopsis shoots and roots. a) Transcript levels in shoots and roots of the *zfp3* mutant and silenced lines ZFP3amiR3/5 and ZFP3amiR3/15. b) Transcript levels of *ZFP3* in Col-0 wild type plants and transgenic plants overexpressing ZFP3 under the control of pCaMV35S promoter. c) Transcript levels in wild type and transgenic plants with estradiol-inducible ZFP3 constructs (XVE-ZFP3h1, XVE-ZFP3h2) under short and long estradiol treatments. Relative transcript levels are shown in log scale. 1 corresponds to the average transcript levels of UBIQ10 and GAPDH2 reference genes.

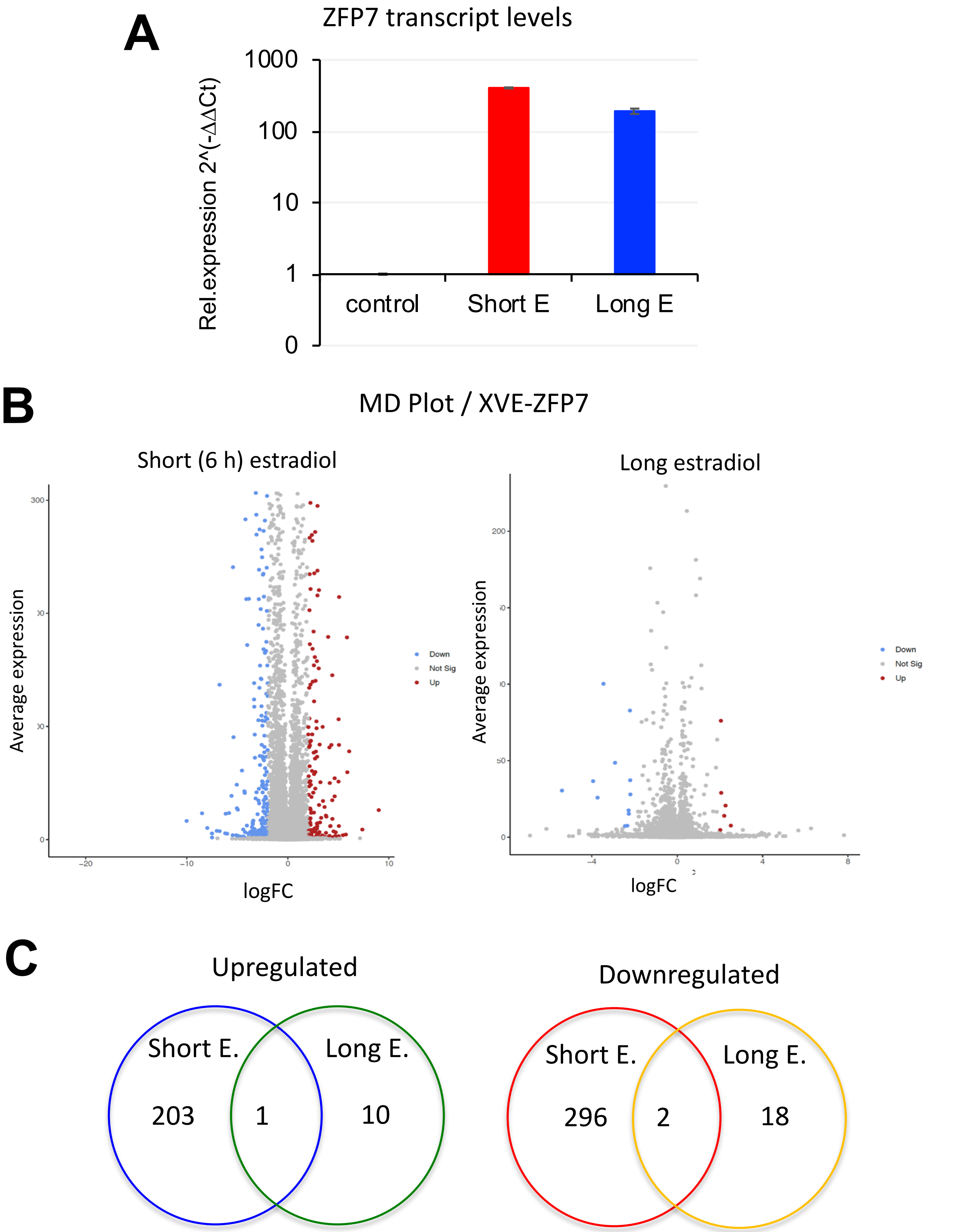

Figure S17. Summary of transcript profiling of ZFP7 overexpressing plants. (A) MD plot of misregulated gene sets from short (6 h) and long (continuous) estradiol-treated plants. Mean Difference plot indicates the log2(Fold Change) values versus mean logCPM expression values, highlighted genes have FDR<0.05. (B) Number of up and downregulated genes (at least 2.5 fold change) in ZFP3 overexpressing plants. Short E. and Long E. indicates 6 h or continuous estradiol treatment of XVE-ZFP3h1 plants.

GO biological process

	Arabidopsis thaliana (REF)	upload_1 (▽ Hierarchy NEW! ?)				
GO biological process complete	#	#	expected	Fold Enrichment	+/-	P value
circadian rhythm	133	12	.96	12.44	+	2.03E-06
↳rhythmic process	133	12	.96	12.44	+	2.03E-06
response to carbohydrate	191	9	1.39	6.50	+	4.65E-02
↳response to organic substance	4560	73	33.07	2.21	+	3.41E-08
↳response to chemical	5308	80	38.50	2.08	+	4.51E-08
↳response to stimulus	9743	121	70.67	1.71	+	1.68E-09
↳response to oxygen-containing compound	3432	61	24.89	2.45	+	5.65E-08
response to cold	651	27	4.72	5.72	+	1.93E-09
↳response to stress	5749	87	41.70	2.09	+	2.01E-09
↳response to temperature stimulus	1309	40	9.49	4.21	+	5.21E-11
↳response to abiotic stimulus	4732	85	34.32	2.48	+	2.48E-13
response to salt stress	711	18	5.16	3.49	+	1.83E-02
response to abscisic acid	1181	29	8.57	3.39	+	4.05E-05
↳response to hormone	2717	44	19.71	2.23	+	1.63E-03
↳response to endogenous stimulus	2748	44	19.93	2.21	+	1.89E-03
↳response to alcohol	1324	30	9.60	3.12	+	1.28E-04
defense response to fungus	927	22	6.72	3.27	+	4.56E-03
↳response to fungus	1183	24	8.58	2.80	+	2.08E-02
↳response to other organism	2673	43	19.39	2.22	+	2.57E-03
↳biological process involved in interspecies interaction between organisms	2687	43	19.49	2.21	+	2.74E-03
↳response to external biotic stimulus	2673	43	19.39	2.22	+	2.57E-03
↳response to biotic stimulus	2676	43	19.41	2.22	+	2.60E-03
↳response to external stimulus	3195	45	23.17	1.94	+	3.63E-02
↳defense response to other organism	2295	36	16.65	2.16	+	4.30E-02
response to wounding	940	22	6.82	3.23	+	5.69E-03
response to water deprivation	1249	28	9.06	3.09	+	4.70E-04
↳response to water	1361	30	9.87	3.04	+	2.30E-04
↳response to salt	1469	31	10.66	2.91	+	3.51E-04
↳response to inorganic substance	2246	38	16.29	2.33	+	4.64E-03
↳response to acid chemical	1394	30	10.11	2.97	+	3.82E-04

Figure S18. GO term categories of genes upregulated by 6 hours of ZFP7 overexpression (short treatment)

	Arabidopsis thaliana (REF)	upload_1 (▽ Hierarchy NEW! ?)				
GO biological process complete	#	#	expected	Fold Enrichment	+/-	P value
cellular response to blue light	42	11	.45	24.52	+	2.33E-08
↳cellular response to light stimulus	183	18	1.95	9.21	+	1.72E-08
↳cellular response to radiation	189	18	2.02	8.92	+	2.82E-08
↳response to radiation	2311	85	24.68	3.44	+	1.04E-20
↳response to abiotic stimulus	4732	119	50.53	2.35	+	2.53E-17
↳response to stimulus	9743	170	104.05	1.63	+	2.04E-11
↳cellular response to abiotic stimulus	274	22	2.93	7.52	+	3.18E-09
↳cellular response to environmental stimulus	274	22	2.93	7.52	+	3.18E-09
↳response to light stimulus	2199	85	23.48	3.62	+	4.09E-22
↳response to blue light	181	20	1.93	10.35	+	1.51E-10
circadian rhythm	133	18	1.42	12.67	+	1.22E-10
↳rhythmic process	133	18	1.42	12.67	+	1.22E-10
photomorphogenesis	88	10	.94	10.64	+	2.73E-04
↳response to red or far red light	409	30	4.37	6.87	+	1.96E-12
regulation of circadian rhythm	66	7	.70	9.93	+	3.66E-02
response to high light intensity	66	7	.70	9.93	+	3.66E-02
photosynthesis, light reaction	165	13	1.76	7.38	+	1.82E-04
↳generation of precursor metabolites and energy	387	17	4.13	4.11	+	4.99E-03
↳photosynthesis	247	18	2.64	6.82	+	1.65E-06
chloroplast organization	238	13	2.54	5.11	+	9.14E-03
↳plastid organization	320	15	3.42	4.39	+	9.68E-03
response to cold	651	29	6.95	4.17	+	7.05E-07
↳response to temperature stimulus	1309	47	13.98	3.36	+	2.01E-09
regulation of post-embryonic development	485	20	5.18	3.86	+	1.55E-03
↳regulation of multicellular organismal development	572	20	6.11	3.27	+	1.73E-02
regulation of DNA-templated transcription	2445	53	26.11	2.03	+	2.73E-03
↳regulation of RNA biosynthetic process	2453	53	26.20	2.02	+	2.92E-03
↳regulation of macromolecule biosynthetic process	2675	56	28.57	1.96	+	4.37E-03
↳regulation of biosynthetic process	3061	70	32.69	2.14	+	3.34E-06
↳regulation of cellular metabolic process	3679	67	39.29	1.71	+	4.19E-02
↳regulation of cellular biosynthetic process	2901	61	30.98	1.97	+	9.10E-04
response to chemical	5308	92	56.69	1.62	+	3.36E-03

Figure S19. GO term categories of genes downregulated by 6 hours of ZFP7 overexpression (short treatment)

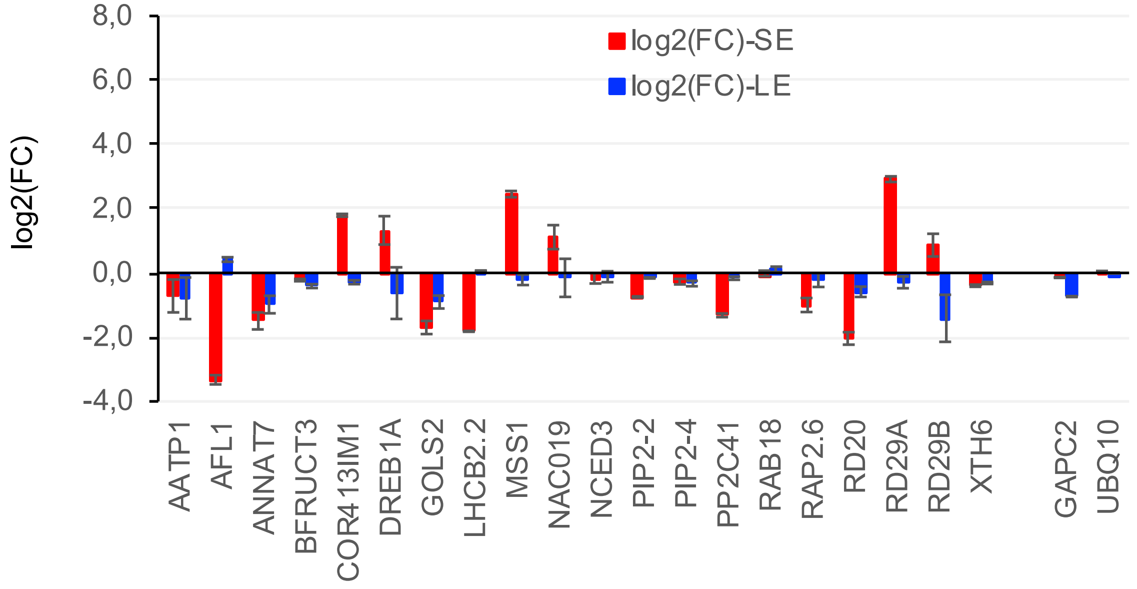

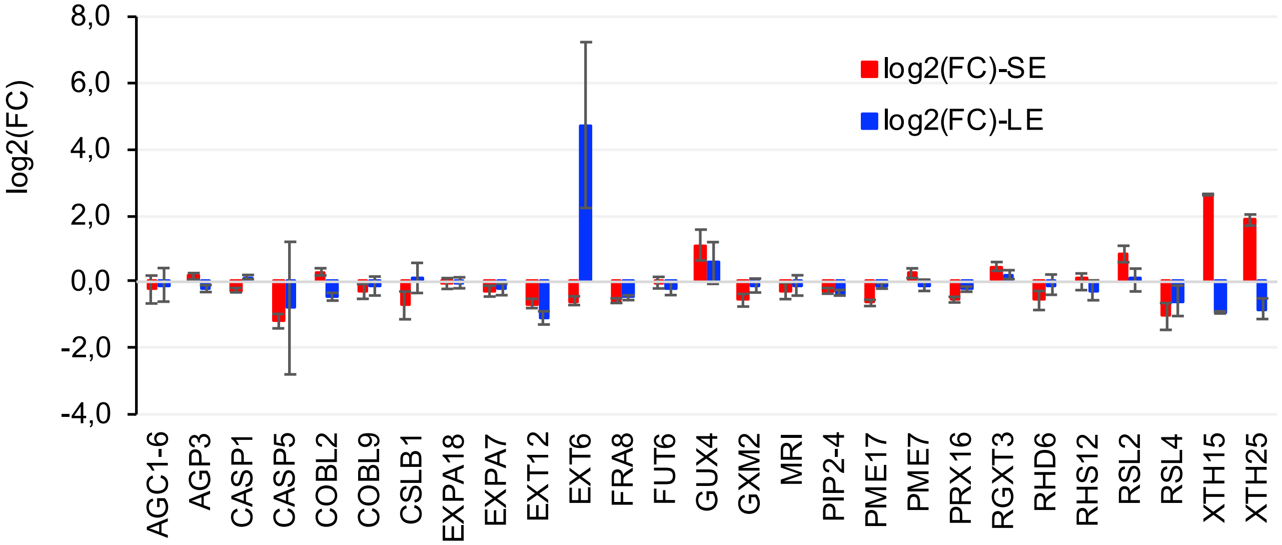

Figure S20. Transcript data of dehydration and ABA-responsive genes and genes implicated in cell wall formation, root hair growth in ZFP7 overexpressing plants. Transcript levels of the genes which were listed in Figures 4 and 5 are shown here. Relative transcript levels are show in log scale (log2(FC), compared to wild type plants (Col-0=0). StdErr indicates standard error. SE: short estradiol treatment, LE: long estradiol treatment.

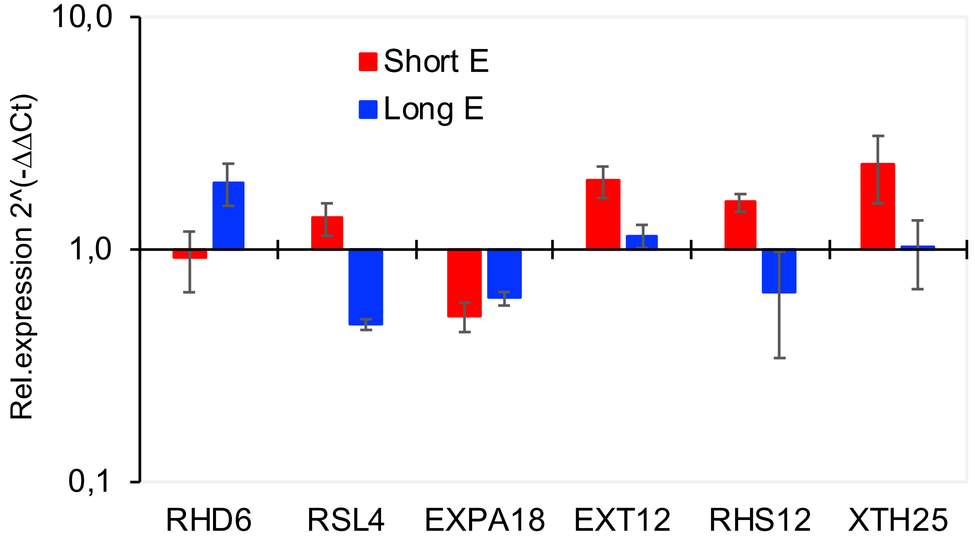

Figure S21. Transcript analysis of selected cell wall and root hair-related genes in ZFP7 overexpressing plants. Relative transcript levels were determined by qRT-PCR. Expression is show in log scale, where 1 corresponds to non-treated control plants (=1). Error bars indicate standard deviation (N=3). These genes were downregulated by ZFP3 overexpression (Figure 5).

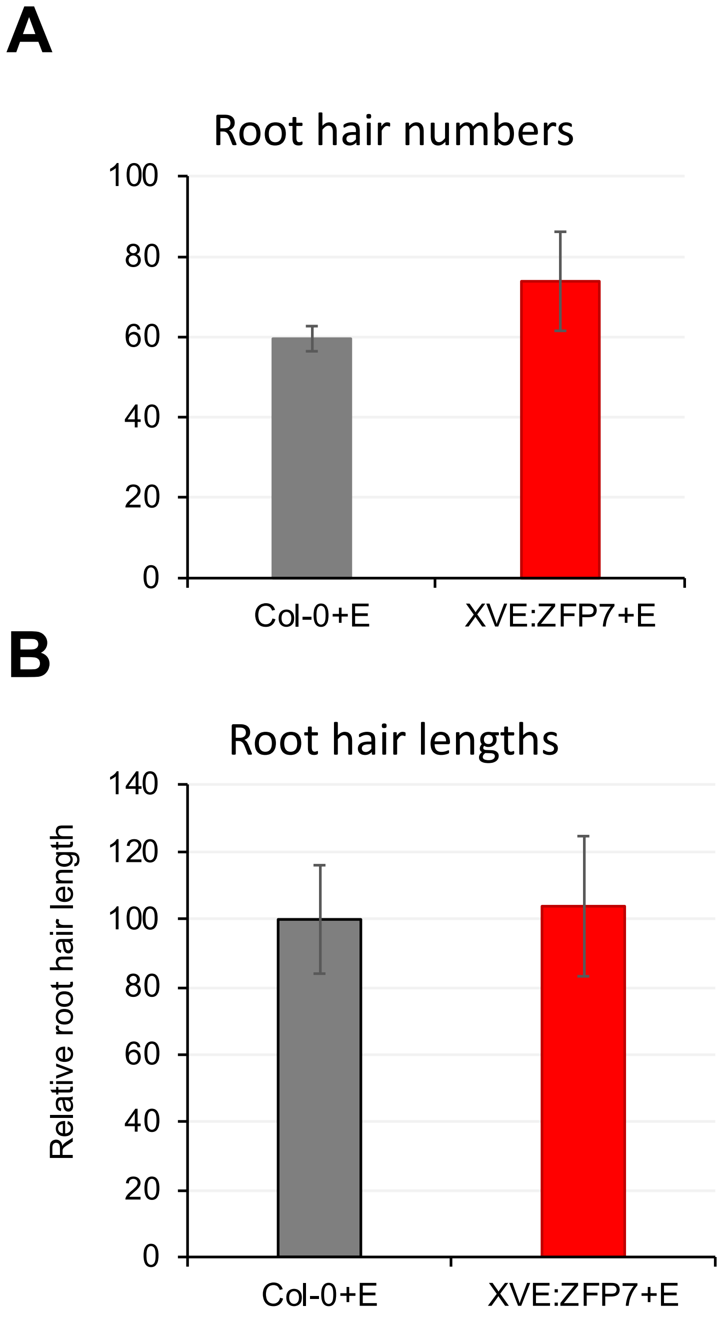

Figure S22. Comparison of root hair formation in Col-0 and ZFP7 overexpressing plants. Seeds were germinated and plantlets were grown on ½ MS medium complemented by 5 μM estradiol. Roots were photographed and number and size of root hairs were determined on 7 days-old plants. Error bars show standard deviation (root hair numbers: n=8, root hair sizes n=160).

Supplemental Method: ImageJ script to measure rosette areas in vitro

```
setBatchMode(true)
path=getDirectory("Choose a Directory");
list=getFileList(path);
for(i=0;i<list.length;i++)     {
        open(path+list[i]);

        run("Lab Stack");
        run("Stack to Images");

        selectWindow("L*");
        run("Grays");
        run("Invert");
        run("8-bit");
        run("Set Measurements...", "mean redirect=None decimal=3");
        run("Measure");
        mean = getResult("Mean");
        run("Subtract...", "value=" + mean);
        run("Clear Results");

        selectWindow("a*");
        run("Grays");
        run("Abs");
        run("8-bit");
        run("Measure");
        mean = getResult("Mean");
        run("Subtract...", "value=" + mean);
        run("Clear Results");

        selectWindow("b*");
        run("Grays");
        run("8-bit");
        run("Measure");
        mean = getResult("Mean");
        run("Subtract...", "value=" + mean);
        run("Clear Results");

    imageCalculator("Average create", "L*","b*");
    imageCalculator("Average create", "Result of L*","a*");
    selectWindow("Result of Result of L*");
    setThreshold(27, 255);
    setOption("BlackBackground", true);
    run("Make Binary");
        run("Close-");
        saveAs("Jpeg", path+list[i]);

        run("Set Measurements...", "area redirect=None decimal=3");
        run("Analyze Particles...", "size=100-Infinity show=Masks display clear add in_situ");
        run("Flatten");
        saveAs("PNG", path+list[i]);

        selectWindow("Results");
        saveAs("txt", path+list[i]);

        while (nImages>0)      {
                selectImage(nImages);
                close();       }       };
if (isOpen("Results")) {
        selectWindow("Results");
        run("Close");  };
if (isOpen("ROI Manager"))     {
        selectWindow("ROI Manager");
        run("Close");
```

Supplemental method: ImageJ script to measure rosette areas in vitro
